# Vaccines for COVID-19: A Systematic Review of Immunogenicity, Current Development, and Future Prospects

**DOI:** 10.3389/fimmu.2022.843928

**Published:** 2022-04-27

**Authors:** Zhan Zhang, Qi Shen, Haocai Chang

**Affiliations:** ^1^Ministry of Education (MOE) Key Laboratory of Laser Life Science & Institute of Laser Life Science, College of Biophotonics, South China Normal University, Guangzhou, China; ^2^Guangdong Provincial Key Laboratory of Laser Life Science, College of Biophotonics, South China Normal University, Guangzhou, China

**Keywords:** COVID-19, SARS-CoV-2, prevention, antigen selection, COVID-19 vaccines

## Abstract

The persistent coronavirus disease 2019 (COVID-19), characterized by severe respiratory syndrome, is caused by coronavirus 2 (SARS-CoV-2), and it poses a major threat to public health all over the world. Currently, optimal COVID-19 management involves effective vaccination. Vaccination is known to greatly enhance immune response against viral infections and reduce public transmission of COVID-19. However, although current vaccines offer some benefits, viral variations and other factors demand the continuous development of vaccines to eliminate this virus from host. Hence, vaccine research and development is crucial and urgent to the elimination of this pandemic. Herein, we summarized the structural and replicatory features of SARS-CoV-2, and focused on vaccine-mediated disease prevention strategies like vaccine antigen selection, vaccine research, and vaccine application. We also evaluated the latest literature on COVID-19 and extensively reviewed action mechanisms, clinical trial (CT) progresses, advantages, as well as disadvantages of various vaccine candidates against SARS-CoV-2. Lastly, we discussed the current viral treatment, prevention trends, and future prospects.

## Introduction

Recent and ongoing viral diseases, caused by novel coronaviruses, are a significant threat to public health worldwide. Governments and researchers of numerous countries are working vigorously to manage past and ongoing epidemics and carry out etiological research. The current coronavirus outbreak was first reported to the World Health Organization (WHO) on 31 December 2019 (1). On January 12, 2020, WHO named this novel coronavirus “2019-nCoV” (2), and on 11 February 2020, WHO formally named the disease “coronavirus disease 2019” or “COVID-19” (3). The same day this coronavirus was also called “severe acute respiratory syndrome coronavirus-2 (SARS-CoV-2)” by the International Committee of classification of viruses (ICTV), due to its similarity with earlier SARS-CoV (3). Globally, as of March 9, 2022, WHO reported over 440 million COVID-19 cases, and the death toll surpassed 6 million deaths (4).

The COVID-19 pandemic prompted an unparalleled worldwide crisis. The coronavirus spread rapidly, destroyed livelihoods of billions of people, and endangered the global economy (5). The reported and confirmed COVID-19 case numbers have not peaked yet and the global condition remains severe. Hence, the United Nations took lead in the global collaborative efforts to fight this disease. Nevertheless, till now, no efficacious anti-COVID19 treatment has emerged (6). This situation prompted numerous countries and regions worldwide to take part in COVID-19 research to eradicate this disease. In particular, the development of highly efficacious anti-COVID-19 vaccine is key to controlling and preventing this pandemic outbreak (7). Herein, we not only introduced the structural characteristics of 2019-nCoV in detail, but also reviewed the latest vaccine types, research progresses, as well as potential and current challenges in the advancement of vaccine research to eradicate this devastating pandemic.

## Structural and Replicatory Features of SARS-CoV-2

**Figure 1:**
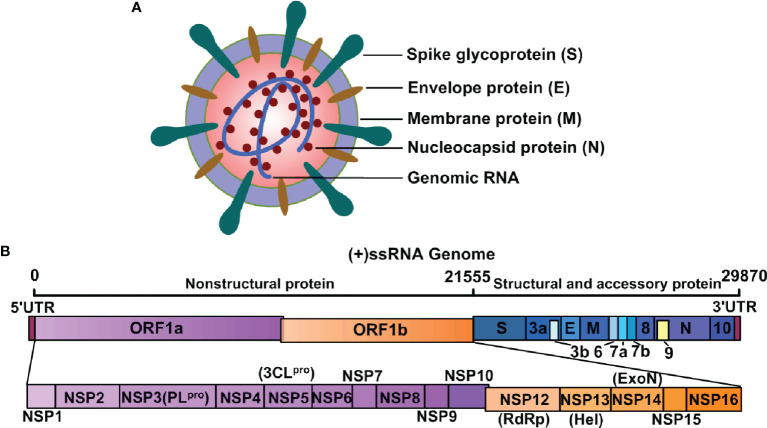
Structure of SARS-CoV-2 and its genomic RNA. **(A)** SARS-CoV-2 is composed of spike glycoprotein (S), envelope protein (E), membrane protein (M), nucleocapsid protein (N), and genomic RNA. These components guide the transcription and translation of its nonstructural, structural, and accessory proteins. **(B)** SARS-CoV-2 is a single-stranded ribonucleic acid (+ssRNA) virus and the genome components encode both structural and nonstructural components of SARS-CoV-2.

**Figure 2:**
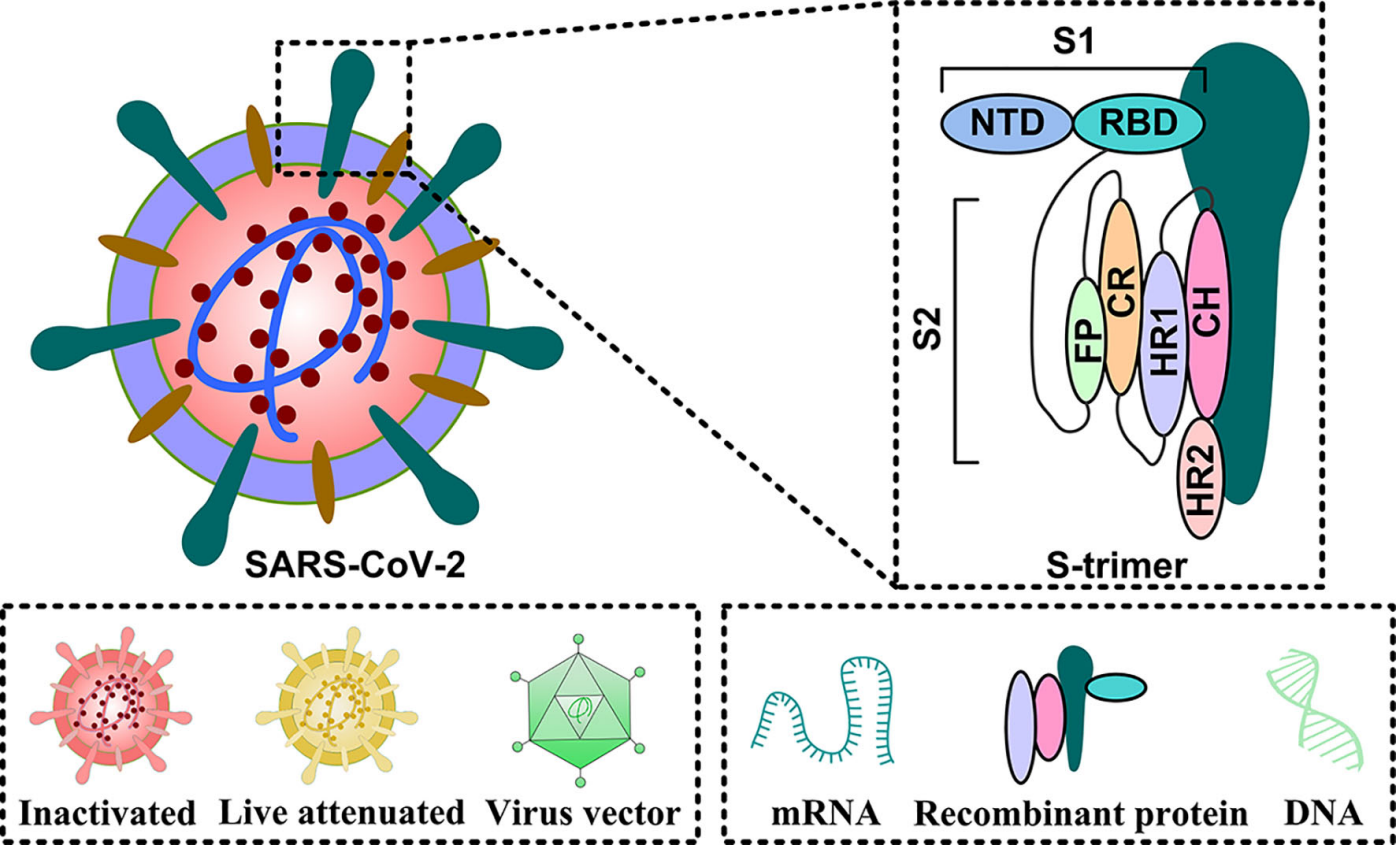
The primary targets of the COVID-19 vaccine candidates. The entire virus can be used as antigen in the following forms: inactivated, live attenuated, and virus vector vaccines. In some cases, functional proteins can also be employed as antigens. These include: mRNA, DNA, and recombinant protein.

The SARS-CoV-2 genetic information is packed inside a single-stranded positive-sense RNA, with variable size (29.8 ~ 29.9 kb), and its genomic composition is similar to other coronaviruses (**Figure 1A**). The open reading frames (ORFs) encode 4 structural [namely, spike surface glycoprotein (S), membrane protein (M), envelope protein (E), and nucleocapsid protein (N)], 16 non-structural (NSP1-16), and some accessory proteins (namely, ORF3a, ORF3b, ORF6, ORF7a, ORF7b, ORF8, ORF9 and ORF10) (8–10) (**Figure 1B**). The S protein has two subunits: N-terminal S1 and C-terminal S2 subunits (11). The S1 subunit is a receptor-interacting domain, with a relatively high structural divergence, and it interacts with the membrane-bound angiotensin-converting enzyme-2 (ACE2) receptor (**Figure 2**). The S2 subunit contains a fusion peptide, and it is responsible for the viral fusion with the host cellular membrane (12). Upon internalization of the bound ACE2 receptor, the host cell type II transmembranal TMPRSS2 serine proteases cleave the S protein to expose the fusion peptide on the S2 subunit and facilitate viral entry into host cells (13). Therefore, most COVID-19 vaccines were developed, based on some aspects of the S protein, to facilitate the disruption of viral entry (14). Among the structural proteins, the M protein is the most abundant. It is a multi-transmembranal protein, with an N-terminal domain (NTD) that is located outside the viral envelope, and a C-terminal domain (CTD) that remains inside the virus. The E protein is the smallest (76-109 amino acids) membranal protein and it possesses ion channel activity (15, 16). In infected host cells, the E protein undergoes massive replication, and a low copy number of the E protein becomes integrated into the viral envelope (16), whereas the majority of E proteins attach themselves to the endoplasmic reticulum-Golgi intermediate compartment (17). In this regard, the functions of both M and E proteins appear to support assembly, formation, and release of viral particles. During infection, the N protein binds with the viral genome at multiple locations, in a nonspecific manner, to protect the RNA as it enters the infected cell and furthers its replication (18, 19). More importantly, the N protein is highly immunogenic and contains antigenic substances that elicit T and B cell responses (20). Being a viral suppressor of RNA silencing (VSR), the N protein antagonizes the antiviral defense of interferon (IFN) and protein kinase R (PKR) (19), which typically arrests protein synthesis during viral replication and transcription. Thus, the N protein may serve as a potential target for vaccines and drug development.

Approximately two-thirds of the SARS-CoV-2 genome contains ORF1a and ORF1b, which encode non-structural proteins. Non-structural proteins are crucial for viral replication and transcription (**Figure 1**). Two cysteine proteases, papain-like cysteine protease (PL^pro^/NSP3) and 3C-like protease (3CL^pro^/NSP5), are indispensable for translated polyprotein processing (21), and can, therefore, be promising targets for antiviral therapy. The RNA-dependent RNA polymerase (RdRp), also known as NSP12, interacts with its co-factors NSP7 and NSP8 to form the replication and transcription complexes (22). NSP13, an NTPase/helicase (Hel), contains a helicase core domain that hydrolyzes all types of NTPs (23), and the zinc-binding and stalk domains are critical for the unwinding of RNA helices, based on the energy released by NTP hydrolysis (24). Another important NSP, NSP14, together with its cofactor NSP10, performs the proofreading of nucleotides by removing nucleotide insertions and mismatches where appropriate (25).

**Figure 3:**
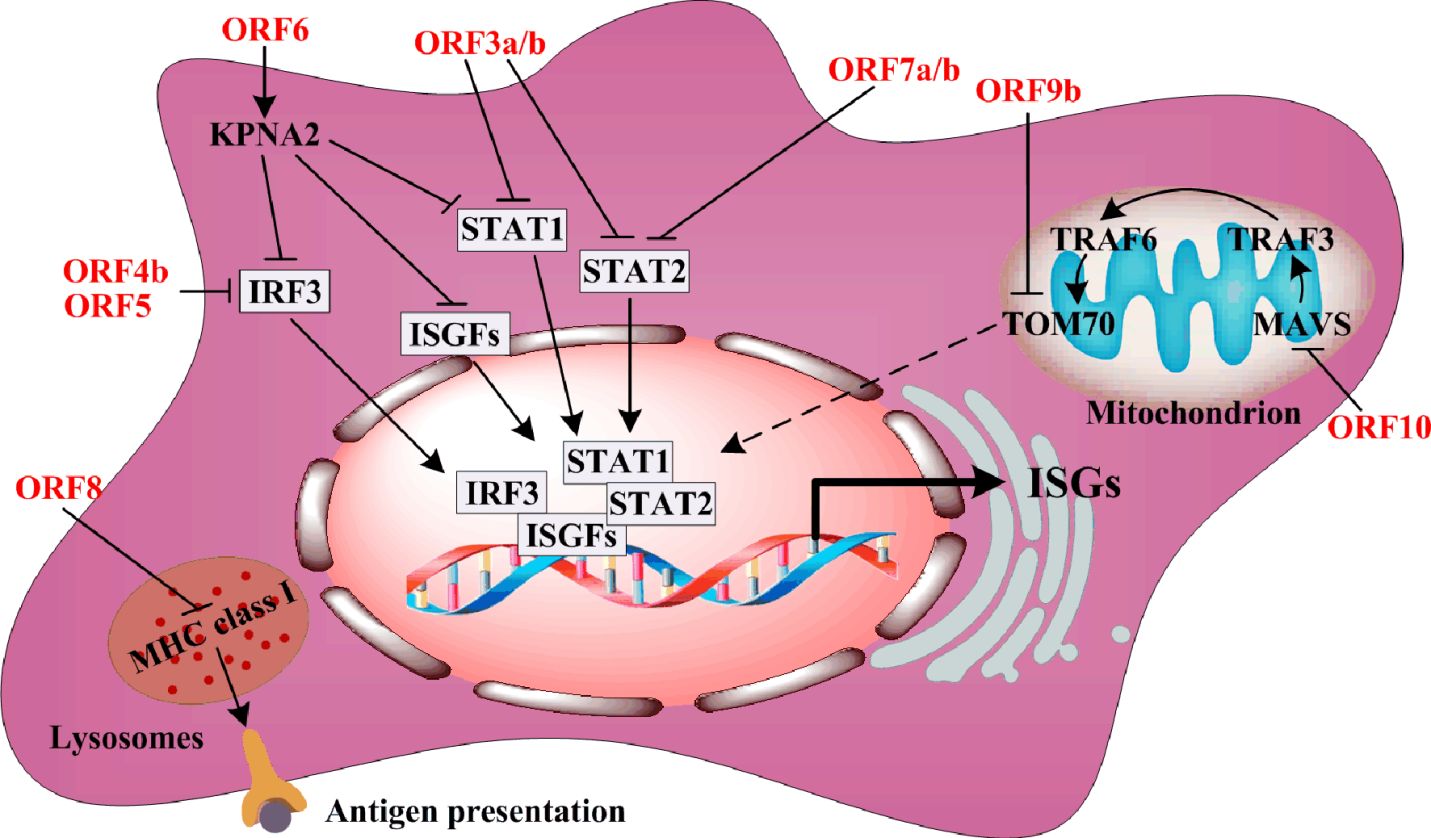
The regulation of ORFs on body immunity.

The accessory proteins usually antagonize type I IFN synthesis or IFN-stimulated gene (ISGs) expression in order to resist host antiviral responses. ORF3a downregulates ISG expression *via* suppression of STAT1 phosphorylation (**Figure 3**). One component of the IFN-stimulated gene factor 3 (ISGF3) harbors STAT1, STAT2, and IRF9, while ORF6 directly blocks IFN-β synthesis by interacting with the nuclear import Karyopherin α2 (KPNA2), thereby inhibiting the nuclear translocation of STAT1, IRF3, and ISGF3 (9, 10). Similar to ORF3a, ORF7a and ORF7b repress STAT2 phosphorylation to downregulate ISGs expression (10). Interestingly, ORF8 interaction with MHC class I molecules in lysosomes disrupts antigen presentation *via* downregulation of their surface antigen expression (26). The mitochondrial localization of ORF9b interacts with translocase of the outer membrane 70 (TOM70), and suppresses IFN-β production by negatively regulating mitochondrial antiviral adaptor protein (MAVS)/TRAF3/TRAF6/TOM70 signaling (27). ORF10 is the smallest accessory protein, and surprisingly, it carries the largest number of immunogenic epitopes (28). Thus, ORF10 may be a potential target for vaccines. However, every SARS-CoV-2 ORF10 variant possesses high frequency mutations, and these mutations need to be monitored during the development of anti-viral therapy.

## Antigen Selection for COVID-19 Vaccines

**Figure 4:**
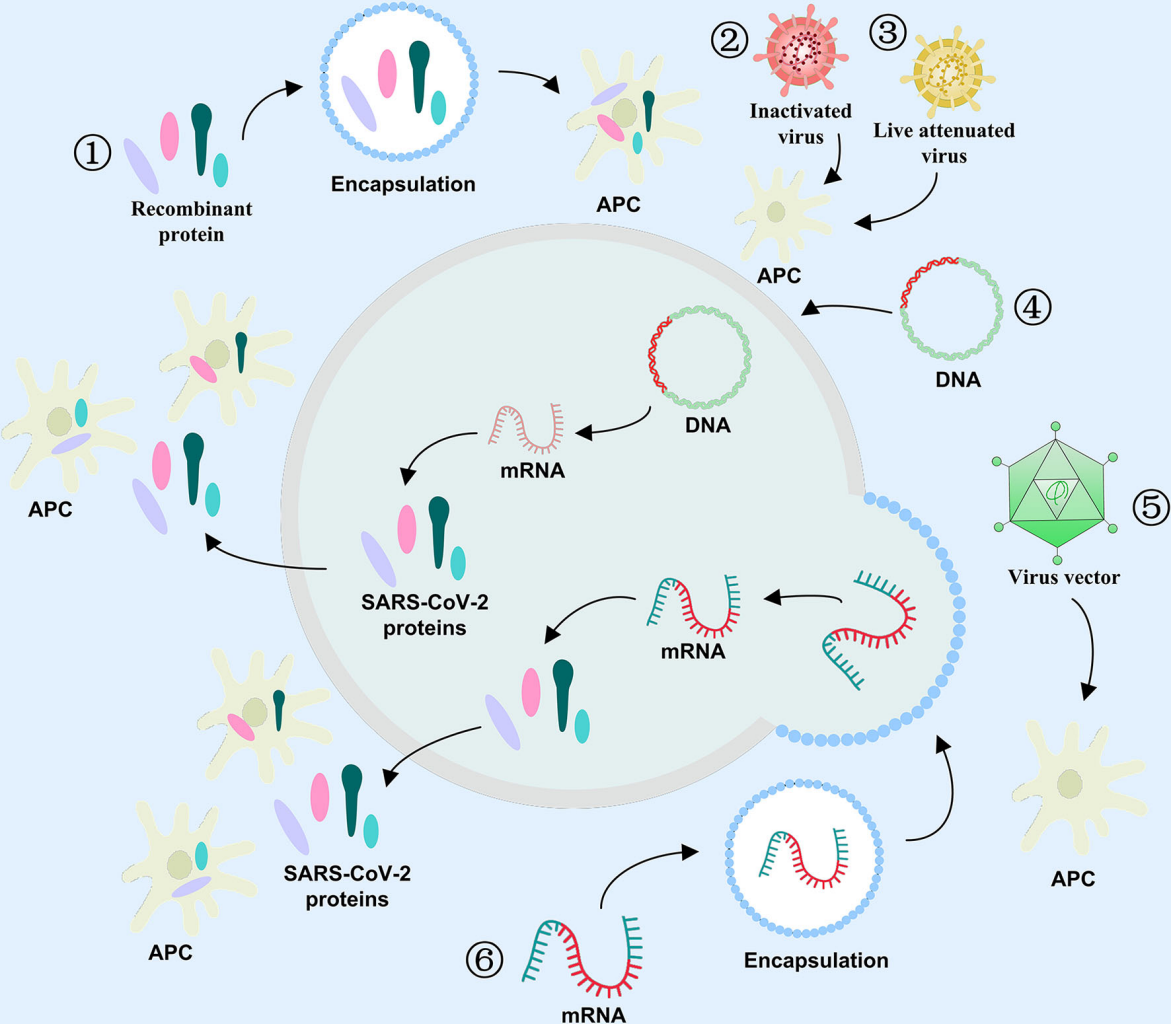
Antigen selection for COVID-19 vaccines. The role of varying antigens in immune response against COVID-19 **①** Recombinant protein, **②** Inactivated virus, **③** Live attenuated virus, **④** DNA, **⑤** Viral vector, and **⑥** mRNA.

Vaccines play a crucial role in the long history of infectious disease prevention, and it does so *via* induction of human herd immunity. Vaccinal antigen is an active vaccine component capable of inducing a specific immune response in the body. Antigens are not only an essential component of vaccines, but are also critical in determining vaccine efficacy. Thus, vaccine development is accompanied by the evolution of vaccine antigen characters. The COVID-19 vaccine antigen can be an attenuated or inactive form of the entire virus or a part of the virus, such as, a protein or sugar. It can also be an mRNA or DNA that induces the host to self assemble vaccine antigens. Inactivated virus, live attenuated virus, and viral vector can directly act on antigen presenting cells (APC), and stimulate immune cells to produce specific antibodies targeting viral antigens (**Figure 4**). Other antigen-specific molecules include protein subunits, RNA, and DNA that indirectly act upon APC in the form of antigenic peptides that undergo modification or packaging within host cells. During the development of an efficacious COVID-19 vaccine, selecting effective antigens will enable the human body to produce humoral or/and cellular immunity, and compel the pathogen to lose its pathogenicity, thereby successfully resisting the virus. Moreover, the aforementioned 2019-nCoV structural proteins, as well as the variety of non-structural proteins, can serve as additional antigen candidates for COVID-19 vaccine development.

The antibody dependent enhancement (ADE) is the enhancement of viral infection through an antibody of low efficacy (29). Owing to its confirmation during transmission of other viruses, including SARS-CoV-1, scientists paid much attention to the ADE effect in the early stages of the SARS-CoV-2 outbreak. Patients with severe COVID-19 often exhibited higher IgG responses, and produced higher total antibody titers, both of which were also associated with poorer outcomes, suggesting a possible ADE effect during COVID-19 infections (30). A study involving the ADE effect in SARS-CoV-2, conducted by the Osaka University, confirmed an increase in antibody dependence after COVID-19 infection (31). More importantly, similar effects were observed in numerous mutated strains, due to the recent emergence of a number of mutated viruses, suggesting that it is unreliable to rely on natural infection to achieve mass immunity (32, 33). Therefore, the biggest challenge facing COVID-19 vaccines remains the ADE effect. From the beginning of the COVID-19 vaccine development, scientists and researchers attempted to identify a SARS-CoV-2 protein, which produces the least ADE effect. Thus far, the ADE effect was not observed in animal studies or human clinical trials. However, this factor requires further investigation, as it involves both vaccine development and treatment. After all, if a potential vaccine, instead of producing neutralizing antibodies, induces the body to form ADE, the outcome can be very dangerous.

### Whole-Viral Antigen

Whole-viral antigen contains all viral components, including proteins, lipids, polysaccharides, nucleic acids, and other components (34). Viral strain separation and extraction are necessary for the preparation of whole-viral antigen. The key steps include eliminating viral activity *via* physical or chemical methods, or reducing viral toxicity *via* artificial mutagenesis (35). Currently, inactivated and attenuated vaccines make up a large portion of COVID-19 vaccines (36). However, it should be noted that the proteins and nucleic acids contained within the whole-viral antigen possess a certain level of immunogenicity, which may induce the body to produce irrelevant antibodies and, thus, reduce the specificity of antibodies against key proteins (37). As such, people often require multiple inoculations to obtain efficient immunity, so the clinical trials (CTs) of whole-viral antigen COVID-19 vaccines are critical for determining the efficiency of such vaccines.

### Spike Protein

The S protein has two subunits S1 and S2 that contribute to viral attachment, fusion, and entry (14). The subunit S1, located at the N-terminal of the virus, harbors the N-terminal (NTD) and receptor-binding domains (RBD) (38). RBD interacts with ACE2 on the host cell membrane, and triggers the merging of the viral envelope with the host membrane (39). Therefore, an effective blockage of the RBD-ACE2 interaction can potentially prevent the SARS-CoV-2 invasion of host cells. Thus, the RBD protein is a potential candidate for the COVID-19 vaccine. The subunit S2 resides in the viral C-terminal. It includes the fusion peptide (FP), connecting region (CR), heptad repeat (HR), and central helix (CH), which primarily regulates the S protein attachment to the host cell membrane whilst mediating the merging of the viral envelope with the host membrane such that the encapsulated virus can enter the cell smoothly and complete the infection (37).

While the S protein is an optimal vaccine target-antigen, the natural S protein has unstable properties (37). Therefore, it is not conducive to the research of S protein function and vaccine development. Therefore, scientists processed the S protein to form S protein trimer, which has enhanced stability. Several studies revealed that a proline substitution of two residues (K986 and V987) increases the S protein (S-2P) stability. This approach was used in some vaccine candidates including Ad26.COV2.S viral vector vaccine, mRNA-1273 vaccine, BNT162b2 mRNA vaccine, and NVX-CoV2373 protein subunit vaccine (40–48). Since the S protein trimer structure is relatively stable, it is among the antigens that are optimal for vaccine generation.

### Other Proteins

Apart from the S protein, other component proteins can also serve as targets of the COVID-19 vaccine (49). The N protein is a helical folding structure formed by the combination of the viral RNA gene chain, and it plays an essential function in viral replication (50). Relative to the S protein, the N protein was shown to be more conserved and stable, with 90% amino acid homology and fewer observed mutations over time (51). Multiple coronavirus N proteins possess high immunogenicity and are expressed in large quantities during infection (52). During an immune response, the intermediate or C-terminal region of the N protein induces antibodies against SARS-CoV-2 (53). The M protein is a transmembranal glycoprotein, and it is distributed on the surface of SARS-CoV-2, where it participates in virus replication (54). The M protein plays a critical role in the structural stability and functional expression of other structural proteins (55). Due to its highly conserved nature, the M protein can also be used as a target antigen for developing SARS-CoV-2 vaccine.

## COVID-19 Vaccines

**Figure 5:**
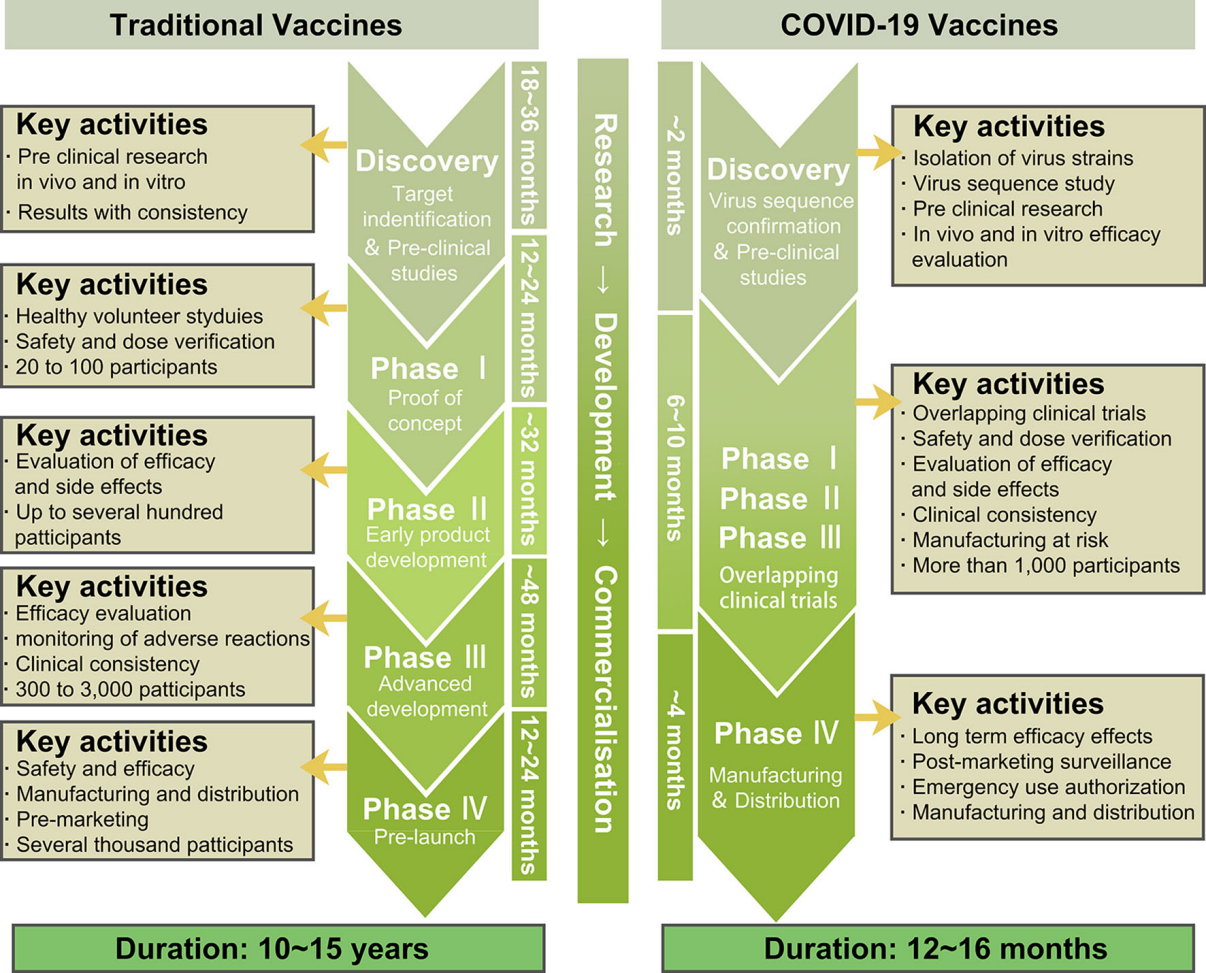
The rapid development of COVID-19 vaccines.

The optimal strategy to ending this pandemic is the development of a highly efficacious anti-COVID-19 vaccine. Therefore, currently, global scientists are actively tackling the research and development of COVID-19 vaccines. The accelerated scientific research and CTs, along with the government-approved emergency use of life-saving COVID-19 vaccines are the main current efforts in combating this pandemic. So far, multiple vaccines have either completed CTs or are in CTs. Compared to other vaccines, the time spent in pre-CT and CT research in developing the COVID-19 vaccine was significantly less. However, this was a tradeoff on the premise of ensuring safety and effectiveness (**Figure 5**).

**Figure 6:**
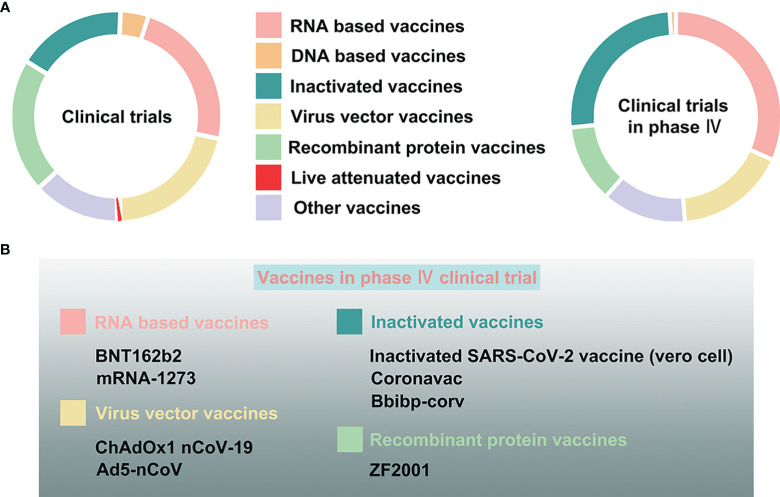
The clinical trials assessing COVID-19 vaccines. **(A)** The proportion of varying vaccine types, namely, RNA-based, DNA-based, inactivated, live attenuated, virus vector, recombinant protein, and other vaccines, in all clinical trials, and especially in phase IV clinical trials. **(B)** The COVID-19 vaccines in phase IV clinical trials.

Currently, scientists all over the world are exploring all possible strategies to develop a highly efficacious vaccine against COVID-19. As mentioned before, the S protein is an optimal candidate protein for vaccine development, since it is the main target of host immune defense (49). Therefore, specific and effective neutralizing antibodies against COVID-19 can be produced that specifically target this early stage of infection. COVID-19 vaccines broke the record in the laboratory experimentation to first-in-human trials timeline (56). As of October 27, 2021, the International CTs Registry Platform (ICTRP) and COVID-NMA, an international initiative working in conjunction with WHO, reported more than 600 CTs examining COVID-19 vaccines (57). Among all CTs, the top vaccine types include traditional vaccines like inactivated and recombinant protein vaccines, as well as some novel vaccines like RNA-based, DNA-based, and non replicating viral vector vaccines, the percentages of which account for 84% of the total CTs (57) (**Figure 6**).

### Inactivated Vaccines

Inactivated viral vaccines are developed by culturing the viruses first, and then inactivating them with heat or chemical agents (6). These vaccines are typically composed of the entire virus, or split viral fragments. Whole virus inactivated vaccine is a classic vaccine technology route (7). Put simply, the virus itself is killed whereas its shell is retained. Therefore, it is unable to induce host cell interference, but it does elicit a strong immune response, based on humoral immunity (58). In response to inactivated vaccines, host immune system can boost antibody production that neutralize and remove pathogenic microbes as well as their toxins so they do not bind target cell receptors and initiate infection. The production of inactivated vaccines is relatively easy. However, their yield depends on *in vitro* viral productivity and need for bio-safe viral production facilities (7).

The efficacy and safety of inactivated vaccines often hold significant advantages. Furthermore, compared to other vaccines, the development cycle of inactivated vaccines is relatively short. Therefore, numerous COVID-19 inactivated vaccines entered into CTs in different countries, including China, India, Turkey, Brazil, Thailand, and Pakistan (3, 9, 10). In particular, about half of the inactivated vaccines entered phase III or IV CTs, and several were approved for marketing, including Sinopharm Beijing Biological Vaccine in China, Sinopharm Wuhan Biological Vaccine in China, and Sinovac Biotech inactivated vaccine in China (**Table 1**) (57). The simultaneous research and development of different inactivated vaccines and CTs provides guarantee for faster production of effective vaccines. Two inactivated vaccines developed in China, namely, the inactivated sars-cov-2 vaccine (vero cell) and the Coronavac, can effectively produce a strong immune response, and significantly lower symptomatic COVID-19 risk (59, 60, 72–75). Since the inactivated vaccine is suitable for long-term preservation and transportation, the China-based vaccine was supplied to multiple countries all over the world (205, 206). However, serum neutralizing activity against multiple SARS-CoV-2 variants of concern (VOCs), including Delta, Beta, and Omicron variants, elicited by the inactivated vaccines is decreased, suggesting that inactivated vaccine protection against mutant strains is weakened and further immunization is needed to deal with the VOCs (61, 76, 207).

**Table 1:** SARS-CoV-2 vaccine candidates in clinical phase III and IV.

Vaccine Type	Vaccine Name	Country/Organization	Targets	Phase	Safety	Efficacy	Reference
Inactivated virus	Inactivated sars-cov-2 vaccine (vero cell)	China	Whole inactivated SARS-CoV-2 with aluminum hydroxide adjuvant	**Phase III**	Serious adverse events were rare.	Treatment of adults with the inactivated SARS-CoV-2 vaccines significantly reduced the risk of symptomatic COVID-19.	(59–71)
				ChiCTR2000034780			
				ChiCTR2000039000			
				NCT04510207			
				NCT04612972			
				NCT04659239			
				NCT04852705			
				PER-051-20			
				**Phase IV**		Protection against VOCs is weakened.	
				ChiCTR2100043907			
				ChiCTR2100046174			
				ChiCTR2100046227			
				ChiCTR2100050589			
				NCT05065892			
				NCT05065879			
Inactivated virus	Coronavac	China	Whole inactivated SARS-CoV-2 with aluminum hydroxide adjuvant	**Phase III**	Immunization with Coronavac in a 0-14 schedule in adults is safe, induces anti-S1-RBD IgG with neutralizing capacity, activates T cells, and promotes the secretion of IFN-γ upon stimulation with SARS-CoV-2 antigens.	The vaccine exhibited a over 60% efficacy on average at preventing COVID-19 illness with favorable safety and immunogenicity profiles.	(72–88)
				NCT04456595			
				NCT04582344			
				NCT04651790			
				NCT04942405			
				**Phase IV**			
				NCT04747821			
				NCT04756830			
				NCT04775069			
				NCT04789356			
				NCT04911790		Protection against VOCs is weakened and further immunization is needed.	
				NCT04953325			
				NCT04962308			
				RBR-9ksh5f4			
				TCTR20210308003			
				NCT04801667			
Inactivated virus	Covaxin	India	Whole inactivated SARS-CoV-2 with Algel-IMDG adjuvant	**Phase III**	Vaccination was well tolerated with no safety concerns raised.	A protective effect of 77.8% against symptomatic COVID-19. Effectively protect against VOCs.	(89–95)
				NCT04641481			
				**Phase IV**			
				CTRI/2021/08/035648			
Inactivated virus	Bbibp-corv	China	Whole Inactivated SARS-CoV-2	**Phase III**	BBIBP-CorV is tolerable and immunogenic in healthy people.	More than 75% of the vaccinators had seroconversion after the first vaccination.	(60, 63, 96–106)
				NCT04560881 NCT04917523 NCT04984408 TCTR20210923013			
				**Phase IV**			
				NCT04863638 TCTR20210910002 NCT05079152 NCT05105295 NCT05104216			
Inactivated virus	QazCovid-in	Kazakhstan	Whole Inactivated SARS-CoV-2	**Phase III** NCT04691908	High safety and potency.	Preliminary results of studies demonstrate efficacy of the vaccine at 96%.	(107, 108)
Inactivated virus	VLA2001	Cooperation between France and Britain	Whole inactivated SARS-CoV-2 with high S-protein density, in combination with two adjuvants, alum and CpG 1018	**Phase III** NCT04864561 NCT04956224	Not reported.	Not reported.	(109, 110)
RNA based vaccines	BNT162b2	Germany	Full-length S protein with proline substitutions	**Phase III**	High safety.	It was well tolerated and could induce neutralizing antibodies.	(111–122)
				NCT04713553 NCT04754594 NCT04800133 NCT04816669 NCT04955626			
				**Phase IV**		Better protection for VOCs.	
				NCT04852861 NCT04951323			
RNA based vaccines	mRNA-1273	America	Full-length S-2P protein	**Phase III**	No safety concerns were identified.	The vaccine showed 94.1% efficacy in preventing SARS-CoV-2.	(42, 112, 123–133)
				NCT04470427 NCT04805125 NCT04806113 NCT04811664 NCT04860297			
				**Phase IV**		Better protection for VOCs.	
				NCT04885907 NCT04952402			
RNA based vaccines	CVnCoV	Germany	Full-length S-2P protein	**Phase III**	Two doses of vaccine were safe.	The vaccine could effectively induce immune response.	(134–139)
				NCT04674189 NCT04838847 NCT04860258			
						CVnCoV exists immune escape for VOCs.	
RNA based vaccines	ARCoV	China	Encoding the RBD of S protein	**Phase III**	Not reported.	Not reported.	(140, 141)
				NCT04847102			
DNA based vaccines	ZyCov-D	India	S protein	**Phase III** CTRI/2021/01/03041-6	Not reported.	The vaccine has 66.6% efficacy from Clinical trials.	(142–144)
DNA based vaccines	Electroporation+ino-4800	America	S1 and S2 subunits	**Phase III** PACTR20211062694-4896	The vaccine showed excellent safety and tolerability.	The vaccine induced a protective immune response.	(145–147)
Recombinant protein vaccines	Recombinant SARS-CoV-2 vaccine (CHO Cell) (ZF2001)	China	RBD-Dimer with alum adjuvant	**Phase III**	Not reported.	Have good tolerance and immunogenicityand be effective effect on neutralization of VOCs.	(148–154)
				ChiCTR2000040153 NCT04646590 NCT05091411 ChiCTR2100050849			
Recombinant protein vaccines	Recombinant SARS-CoV-2 vaccine (Sf9 Cell)	China	RBD with alum adjuvant	**Phase III**	Not reported.	Not reported.	(155, 156)
				NCT04887207 NCT04904471 PACTR20210384538-1761			
Recombinant protein vaccines	NVX-CoV2373	America	S protein with Matrix-M adjuvant	**Phase III**	High safety.	The overall effectiveness of the vaccine is more than 80%.	(41, 157–160)
				NCT04583995 NCT04611802			
Recombinant protein vaccines	Nanocovax	Vietnam	Recombinant S protein with alum adjuvant	**Phase III**	Not reported.	Not reported.	(161)
				NCT04922788			
Recombinant protein vaccines	MVC-COV1901	America	Recombinant S protein with CpG 1018 and alum adjuvants	**Phase III**	MVC-COV1901 has a good safety profile.	The vaccine could elicit promising immunogenicity responses.	(162–165)
				NCT05011526			
				**Phase IV**			
				NCT05097053			
Recombinant protein vaccines	EpiVacCorona	Russia	Peptide antigens of SARS-CoV-2 proteins with alum adjuvant	**Phase III**	Not reported.	Not reported.	(166)
				NCT04780035			
Recombinant protein vaccines	CIGB-66 (RBD/aluminium hydroxide)	ICGEB	RBD with aluminum hydroxide adjuvant	**Phase III**	High safety.	High efficiency.	(167)
				RPCEC00000359			
Recombinant protein vaccines	Razi Cov Pars	Razi Vaccine and Serum Research Institute	Recombinant S protein	**Phase III**	Not reported.	Not reported.	(168)
				IRCT2020121404970-9N3			
Recombinant protein vaccines	FINLAY-FR-2 anti-SARS-CoV-2 Vaccine	Instituto Finlay de Vacunas	RBD with adjuvant	**Phase III**	Not reported.	Not reported.	(169, 170)
				RPCEC00000354 IRCT2021030305055-8N1			
Virus vector vaccines	ChAdOx1 nCoV-19	Britain	Chimpanzee adenovirusvectored vaccine (ChAdOx1) expressing S protein	**Phase III**	ChAdOx1 nCoV-19 has an acceptable safety profile.	ChAdOx1 nCoV-19 is efficacious against symptomatic COVID-19.	(171–179)
				ISRCTN89951424 NCT04516746 NCT04536051 NCT04540393			
				**Phase IV**			
				NCT04760132			
Virus vector vaccines	Ad5-ncov	China	Recombinant replicationdefective human type 5 adenovirus (Ad5) expressing S protein	**Phase III**	High safety.	Ad5-nCoV was well tolerated and could elicit neutralizing antibody responses.	(180–187)
				ChiCTR2100044249NCT04526990 NCT04540419			
				**Phase IV**			
				NCT04892459 NCT04952727			
Virus vector vaccines	Ad26.COV2.S	America	Recombinant replication-incompetent adenovirus serotype 26 (Ad26) vector encoding full-length S protein	**Phase III**	High safety.	The vaccine protected against symptomatic Covid-19 and asymptomatic SARS-CoV-2 infection.	(44, 45, 188–193)
				NCT04505722 NCT04614948 NCT04838795 NCT05028257			
				**Phase IV**			
				NCT05030974			
Virus vector vaccines	Gam-COVID-Vac	Russia	Recombinant Ad26 and recombinant Ad5 encoding full-length S protein	**Phase III**	High safety.	Well tolerated 91.6% efficacy against COVID-19.	(194–201)
				NCT04530396 NCT04564716 NCT04642339 NCT04656613			
Virus vector vaccines	Sputnik light vaccine	Russia	Recombinant Ad26 vector carrying the gene for SARS-CoV-2 S glycoprotein	**Phase III**	Sputnik light vaccine has a good safety profile.	Strong humoral and cellular immune responses both in seronegative and seropositive participants.	(202–204)
				NCT04741061 PACTR20210460157-2565			

Covaxin is the first indigenous anti-COVID-19 vaccine developed in India (208), where the epidemic is still very serious. Coupled with various COVID-19 mutations, it is difficult for some regions to cope with the impact of the epidemic (209). However, India made considerable contributions to the prevention and control of the epidemic (210). Studies revealed that the inactivated vaccine Covaxin effectively neutralizes a variety of recently emerging variants of SARS-CoV-2 (89, 90). In addition, Covaxin Booster could effectively protect against VOCs, including Delta and Omicron variants (91, 92). Covaxin promotes and enhances the response of cytokines and chemokines (211). Following vaccination, the innate and adaptive immune responses of vaccine recipients are also strongly activated (211). In a large phase III CTs, the vaccine was found to have a 77.8% protective effect against symptomatic COVID-19, with no major side effects (93). However, it is worrying that the Covaxin vaccine lacks some reports on the detailed analysis of vaccine development and any potential limitations in research design (208).

Inactivated vaccines do not cause disease, even in immunosuppressed individuals, but because they do not have a durable and long-lasting immune responses, they usually require repeat or enhanced doses, as well as adjuvants, such as, aluminum salt to be added to the vaccine.

### Live Attenuated Vaccines

Live attenuated vaccine employs viruses that undergo multiple treatments or mutations to reduce pathogenecity, without diminishing immunogenicity (11). As a result, inoculation with live attenuated vaccine will not result in disease. Instead, the attenuated pathogen will continue to grow and replicate within the host body, and trigger host immune response. This aids in the acquisition of long-term or life-long protection (12). Compared to inactivated vaccines, live attenuated vaccines elicit stronger immunity and longer action time (212). However, these vaccines may not be suitable for people with compromised immune systems. Therefore, safety can be a major issue with potential risk of disease. The TMV-083 vaccine employs the measles pathogen as a vector and expresses the SARS-CoV-2 S protein antigen (13). Thus far, three live attenuated vaccine candidates, namely COVI-VAC, MV-014-212, and DelNS1-nCoV-RBD LAIV, completed phase I CTs examining safety and immunogenicity (57).

### RNA-Based Vaccines

Although traditional vaccines have successfully prevented multiple diseases, in face of acute outbreaks like this new coronavirus, the development and production cycle of traditional vaccines are too time consuming to meet the needs of epidemic control (213, 214). Therefore, we urgently need a more effective and universal vaccine development platform, and mRNA vaccines are a potential solution to circumventing long vaccine production time.

The mRNA vaccine is a nucleic acid sequence that encodes a specific antigen protein synthesized *in vitro* prior to injection into the human body. Once injected, the specific protein is synthesized within the body, and induces a strong cellular and humoral immune response. This, in turn, produces specific antibodies and exerts immune protection (215). For mRNA vaccines, there is no need to inject pathogenic proteins into the body. Instead, the mRNA is introduced for translation and expression of pathogenic proteins by the vaccine recipient. This approach greatly reduces the body’s adverse reactions and achieves high-efficiency immune effects. Currently, two primary mRNA vaccines are employed against infectious pathogens, including self-amplifying or replicon RNA and non-replicating RNA vaccines (216). Both vaccines produce antigen targets using the body’s own translation mechanism, and then trigger an adaptive immune response within the body (217). Self-amplifying mRNA (SAM) vaccines employ an alpha viral genome, with intact RNA replication machinery, and structural information substituted with mRNA encoding the antigenic protein (218). Since the antigen-encoding RNA can replicate inside the body, the SAM platform facilitates massive antigen synthesis from a relatively small dose of vaccine. Non-replicating mRNA vaccines are artificially transcribed regions of whole mRNA encoding antigenic proteins. This includes the 5’ and 3’ untranslated regions, and the poly(A) tail that adds stability to the mRNA and aids in transcription (219, 220). Other non-replicating mRNA vaccines include plasmid DNA or other DNA fragments that contain the open reading frame of target protein, and are synthesized *in vitro* (221, 222).

Pre-CT research on anti-COVID-19 mRNA vaccines has rapidly increased in recent times, and some have entered human CTs. Once Chinese scholars revealed the COVID-19 gene sequence for the first time, the first COVID-19 vaccine candidates were mRNA-1273, developed by National Institutes of Health (NIH), and Moderna, funded by the Coalition for Epidemic Preparedness Innovations (CEPI) (223). mRNA-1273 is a newly developed nanoparticle (LNP)-encapsulated mRNA vaccine encoding the prefusion stabilized full-length S protein of SARS-CoV-2 (36). In an mRNA-1273 phase I trial, the vaccine exerted a powerful neutralizing antibody titer after two vaccinations (42). On day 43, the SARS-CoV-2 neutralizing activity was observed in all assessed participants, and the antibody levels were comparable to a patient who recuperated from a new crown. Moderna recently published data on an ongoing phase III CT of the mRNA-1273 vaccine. The aforementioned trial included 30,000 subjects of varying ages (between 18 and 85 years old) (123). All participants were arbitrarily separated into two groups receiving two doses of either 100 μg mRNA-1273 or saline placebo, 28 days apart. The protective effect rate of the mRNA-1273 vaccine was 94.1%. They reported only 11 COVID-19 cases among the vaccinated participants, and 185 cases among the placebo cohort. Based on this study, this vaccine not only exerted a good protective effect, but was also relatively safe, with minimal complications (123). In a phase IV CT, involving 120 samples, the immunogenicity of a third dose of the mRNA-1273 vaccine in transplant recipients was shown to be significantly higher than the placebo (224).

Meanwhile, Biotech and Pfizer developed two COVID-19 lipid nanoparticle-formulated, nucleoside-modified RNA vaccination candidates: BNT162b1 and BNT162b2. In the pre-CT and CTs, BNT162B1 and BNT162B2 demonstrated significant safety and immune response advantages (48, 225). Upon extensive review of the phase I/II pre-CT and CT data, including consultations with the US Food and Drug Administration Center for Biologics Evaluation and Research (CBER) and other global regulatory agencies, the vaccine manufacturers selected 30 μg BNT162b2 in a two-dose immunization regimen to proceed to the crucial phase III safety and efficacy assessment. In the phase III CT, 43,448 healthy individuals were arbitrarily selected to receive injections: 21,720 with BNT162b2 and 21,728 with placebo (111). In the sample population, a total of 170 COVID-19 cases emerged, after at least 7 days of booster immunization. Among these, only 8 cases were among vaccinated individuals, and 162 cases among the placebo cohort. Therefore, the BNT162b2 protection rate was 95.0%. A survey of nearly 600,000 vaccinators further revealed that the two doses of the BNT162b2 vaccine prevented 92% of infections and 94% of symptomatic COVID-19 (226). Vaccination with mRNA-1273 or BNT162b2 vaccine provided better protection, but the effect of mrNA-1273 vaccine seemed to be better for α and δ variants (112). Due to the urgent need of the epidemic, two COVID-19 mRNA vaccines (mRNA-1273 and BNT162b2) were approved by the FDA for their versatility and rapid development advantages.

CV2CoV, an enhanced mRNA-driven SARS-CoV-2 vaccine candidate, accelerates virus neutralizing antibody production, and positively regulates immune protection in rodents (227). Research demonstrated that CV2CoV supports elevated protein levels and enhanced immunogenicity in a rat pre-CT (228). In a pre-clinical study, CV2CoV was also able to achieve a stronger antibody neutralization of variants, including the beta, delta, and lambda variants (229). According to a recent phase III CT assessing the efficacy and safety of the CVnCoV SARS-CoV-2 mRNA candidate vaccine, CVnCoV effectively prevents COVID-19 of any severity and has an acceptable safety profile (230). However, CVnCoV exists immune escape for SARS-CoV-2 variant (134, 135). Therefore, vaccination needs to be strengthened to deal with the weakened immune response and the emerging SARS-CoV-2 variants.

Compared to traditional vaccines, the difficulty and key technology of the mRNA vaccine development mostly lies in its modification and delivery system (217, 231). In fact, mRNA synthesis and modification can vastly improve its molecular stability, while preventing degradation. The delivery system selection can also improve the efficiency of mRNA entry into human cells, to produce antigen, and stimulate an immune response within the host. Both Moderna and BioNTech chemically modified their mRNA vaccines, replacing Uridine (U) with Pseudouridine (ψ), which reduced immunogenicity and increased stability of the mRNA (232). In contrast, CureVac, one of the three giants in mRNA vaccine research and development, employed unmodified uridine to enhance mRNA translation through sequence optimization and selection of untranslated regions (UTRs), perhaps to circumvent patent issues related to mRNA molecular modification, thus, resulting in high immunogenicity, low dose, and poor effect (233, 234). In addition, lipid nanoparticles (LNPs) are the current most advanced and mainstream mRNA delivery system (235). Their high entrapment efficiency protects mRNA from degradation, and the easy fusion of liposomes with recipient cells improves delivery efficiency. The delivery mechanism of LNPs is mainly through the combination of cationic liposomes with negatively charged mRNA to form complexes with particle size less than 200nm, and then enter cells through endocytosis (236). LNPs delivery system was used for all COVID-19 mRNA vaccines. BioNTech and Moderna’s COVID-19 mRNA vaccines use ionizable lipids ALC-0315 and SM102, respectively (237). The common auxiliary lipids were DSPC and cholesterol. Due to the complex relationship between patent protection and license transaction of COVID-19 mRNA vaccines, the LNP delivery system is a major focus of competition. However, none of these technologies have been commercialized thus far.

The biggest advantage of such vaccines is that they can be produced completely *in vitro*. However, it is unclear what challenges may surface during large-scale production, long-term frozen storage, and in vaccine stability. In addition, since the drug is administered by injection, it is unlikely to induce a strong mucosal immune response.

### DNA-Based Vaccines

DNA vaccine, yet another type of nucleic acid vaccine, is made from DNA encoding antigen protein. Unlike traditional vaccines, DNA vaccines are designed to inject a specific naked DNA code of the pathogen directly into the human body (219, 238). Once inside, it can be transcribed into mRNA in the nucleus and translated into antigen in the cytoplasm, thereby inducing the body to produce an immune response (239). Thus, vaccinated individuals obtain corresponding immune protection and disease prevention capabilities (240). Interestingly, its rapid technological development may create a whole new generation of immunologic tools. Multiple DNA vaccines are currently under development, including malaria, influenza, rotavirus, HIV, and so on. Furthermore, many of them have already entered CTs (241–243). DNA vaccination offers more potential advantages, compared to traditional vaccines, including stimulation of the B and T cell immune responses, stabilization of the vaccine, avoidance of any infectious agents, and ease of large scale production (244). More importantly, a large-scale COVID-19 vaccination is under rapid execution around the world, and the “stability” that determines whether a vaccine is convenient for transportation, and storage is a major indicator of vaccine feasibility (245). Compared to RNA vaccines, DNA vaccines can be stored for a longer period of time at the same refrigeration temperature, or even for a longer time at room temperature (246). Once successfully developed and put into use, this will likely bring great convenience to the mass production of vaccines. However, DNA vaccines usually exhibit low immunogenicity, and must be inoculated through delivery devices (such as, electric perforators) to be effective, which also limits their use.

Due to the COVID-19 pandemic outbreak, numerous DNA vaccine candidates entered CTs. According to the information from ICTRP, three of them entered phase III CTs, namely INO-4800, AG0302-COVID19, and, ZyCoV-D. INO-4800, developed by INOVIO Pharmaceuticals, is a DNA vaccine candidate for the prevention of the new coronavirus (243, 247). After the new coronavirus gene sequence was released, INOVIO employed a proprietary DNA drug platform to quickly design the INO-4800 vaccine (247). Pre-CT revealed that INO-4800 strongly induces SARS-CoV-2 specific antibody and T cell responses in mice and guinea pigs, hence, it was quickly approved for CTs (248). Based on the phase I CT results, INO-4800 is immunogenic in all subjects, and effectively produces humoral immune and/or cellular responses (145). Currently, the INO-4800 vaccine is simultaneously in both phase II/III CTs, and its safety and effectiveness reports are worthy of attention. AG0301-COVID19, a DNA vaccine developed by Osaka University/AnGes/Takara Bio, expresses the full-length S protein upon host cell entry. In a pre-CT, AG0301-COVID19, with an aluminium-containing adjuvant, strongly stimulated neutralizing antibody production, and enhanced T cell responses in rats, with no toxic response to body organs. Yet another COVID-19 DNA vaccine candidate is AG0301-COVID19, also developed by the Osaka University/AnGes/Takara Bio. The safety and effectiveness of the AG0301-COVID19 vaccine was examined in phase I/II CTs, but the results are not yet reported (243). It is worth noting that the phase III CTs are in progress at the present time. Recently, India urgently authorized and approved the listing of a DNA COVID-19 vaccine ZyCov-D that consists of a DNA plasmid vector harboring the S protein genetic code. A pre-CT study revealed that ZyCov-D elicits marked antibody and Th-1 responses, as demonstrated by augmented IFN-c expression (249). In a phase I CT, the ZyCov-D vaccine, which is administered by pressing a needle-free device on the skin, was reported to be safe, well tolerated, and immunogenic (142). The device creates a tiny stream of high-pressure liquid that pierces the skin surface, thus causing less pain for the recipient. ZycoV-D is currently conducting a large-scale phase III CT in India, involving tens of thousands of subjects, but the data is not public yet. However, due to its high efficiency and safety, India urgently authorized the application of ZycoV-D. Thus far, this is not only the first DNA vaccine for COVID-19, but also the first DNA vaccine in the world. Till date, no DNA vaccine completed phase III trials or received approval.

### Recombinant Protein Vaccines

Recombinant vaccine is a virus or protein, produced by modern genetic engineering technology, which can induce immune response in human or animal host to achieve disease prevention or treatment (12). There are two types of vectors: bacteria and virus. The primary mechanism of this vaccine is to employ genetic engineering to introduce and express pathogenic antigen encoding genes in adenoviruses (46). The first step is to identify the gene that encodes the target antigenic protein, for example, the viral S protein (5, 12). The following step involves fusion of this gene with the adenovirus, prior to introduction into human body. Once inside, the encoded gene synthesizes the S protein and induces a strong humoral and cellular immunity that encourages the body to produce target-specific antibodies (3, 5).

Compared to traditional vaccines, recombinant vaccines are not only easier to produce, but also more effective or widespread. Since the pandemic outbreak, the viral recombinant subunit protein vaccine is, for the first time, being jointly developed by cooperative enterprises. The recombinant tandem-repeat dimeric RBD-based protein subunit vaccine (ZF2001) is a recombinant protein vaccine, made from a prefusion-stabilized spike trimer of SARS-CoV-2, and combined with aluminium hydroxide and CpG 7909, prior to expression and purification in Chinese hamster ovary (CHO) cells (151). Immunogenicity studies revealed that the recombinant new coronavirus vaccine (CHO cell) triggered a strong neutralizing antibody response and considerable CD4^+^ T cell responses in both mice and non-human subjects (151). Importantly, the candidate vaccine significantly lowered viral loads and lung inflammation in SARS-CoV-2-infected golden Syrian hamsters (22). According to the phase I and II CT data, the protein subunit vaccine ZF2001 has good tolerance and immunogenicity (148). Importantly, ZF2001 presented effective effect on neutralization of the antisera to SARS-CoV-2 variants including Delta (149, 150). At present, a large-scale phase III CT was launched in China, Uzbekistan, Pakistan, Ecuador, Indonesia and other countries to evaluate the safety and effectiveness of ZF2001. Since the ZF2001 phase III CT is progressing smoothly, it has already received approval for emergency use in China. This makes this vaccine the first virus recombinant subunit protein vaccine to be approved for clinical use in the world.

In conclusion, the key advantage of these vaccines is that they can be produced without employing live viruses, and they possess considerable market production value. However, this kind of vaccine also has certain disadvantages. The S protein is relatively difficult to express, which may affect the yield. In addition, the selection of a purification method may affect the vaccine-triggered immune response.

### Virus Vector Vaccines

Virus vector vaccine, another kind of nucleic acid vaccine, uses non-toxic viruses as vectors. Genes that encode pathogenic antigens are cloned into non-replicating or replicating viral vectors (such as, adenoviruses). The injected pathogenic gene produces corresponding pathogenic protein fragment that induces an immune response to the target pathogen. Since the viral vector itself promotes an immune response, it produces a stronger response than a simple nucleic acid sequence from the target pathogen. Due to the advantages of high titer, reduced pathogenicity, enhanced transduction efficiency, widely infected tissues, and no host cell genome integration, adenovirus vectors are commonly used in experimental and clinical research, including fields of gene transduction, gene therapy, vaccination, and oncolytic therapy.

Being a non-replicating viral vector, a replication-defective human type 5 adenovirus, encoding the SARS-CoV-2 S protein (Ad5-nCoV), developed by the Chinese People’s Liberation Army Academy of Military Sciences and CanSino Biologics, is the first vaccine candidate to enter CT for COVID-19 vaccination in the world. Pre-CT research revealed that a single dose of Ad5-nCoV offers full protection to murine upper and lower respiratory tracts against the SARS-CoV-2 infection (180). It was also confirmed that a single dose of Ad5-nCoV protects ferret upper respiratory tracts against wild-type SARS-CoV-2 infection (180). In a phase I CT, an aerosolized booster vaccination with Ad5-nCoV at 28 days after the first intramuscular injection induces strong IgG and neutralizing antibody responses (181). Single-peripheral blood mononuclear cell RNA sequencing of samples from the COVID-19 vaccine trial participants revealed that the cellular immunity, cell type-specific IFN, and humoral immunity responses with SARS-CoV-2-specific antibodies were enhanced. This indicates that Ad5-nCoV is a promising vaccine candidate for COVID-19 (250). In addition, the percentage of neutralizing antibodies induced by the Ad5-nCoV vaccine in individuals with COVID -19 was higher than in individuals without COVID -19 (251). Phase II CT revealed that Ad5-nCoV was safe and generated a massive immune response in most recipients following a single dose of vaccine (252). At present, Ad5-nCoV is in phase III CTs and received approval for emergency use in multiple countries.

ChAdOx1 nCoV-19, co-developed by the Oxford University and AstraZeneca, also demonstrated satisfactory safety profile and efficacy, and homologous booster shots enhanced antibody responses in phase I/II CTs (253, 254). The ChAdOx1 nCoV-19 vaccine not only possesses immunogenicity, and induces strong humoral and cell-mediated responses in mice, but also effectively prevents SARS-CoV-2 pneumonia in rhesus monkeys (255). Additionally, the vaccine is tolerant and promotes neutralizing antibody and antigen-specific T cell production against the SARS-CoV-2 S protein (256). Based on the phase III CT study, ChAdOx1 nCoV-19 tolerance is enhanced in older adults, relative to younger adults. In addition, a booster shot induces comparable immunogenicity in all age groups (171).

In addition, the Ad26 vector-based COVID-19 vaccine (Ad26.COV2.S) is a non replicating viral vector vaccine that encodes a prefusion-stabilized SARS-CoV-2 spike immunogen, and it induces effective humoral and cellular immune responses (257), and enhances immunogenicity and reactivity of booster vaccinations (258). Pre-CT revealed that Ad26.COV2.S protects against SARS-CoV-2, and develops better immunogenicity in rats, as well as adult and elderly rhesus monkeys, suggesting that it may be a potential vaccine candidate for COVID-19 (43, 46, 259). In multiple CTs, the candidate vaccine Ad26.COV2.S was found to be both safe and immunogenic in both younger and older adults (44, 260, 261). Considering the safety and effectiveness of pre-CT, several phase III CTs involving Ad26.COV2.S are currently underway. The outcome of Ad26.COV2.S on infection and transmission is yet to be released. Gam-COVID-Vac and Sputnik light vaccine, two additional non-replicating viral vector vaccines, employ the recombinant adenovirus type 26 (rAd26) vector harboring the SARS-CoV-2 S protein genetic code, and were developed by Russia. Single immunization with the Ad26 vector vaccine expressing the stable SARS-CoV-2 spike protein elicits interaction and neutralizing antibody reactions. The Ad26 vaccine safeguards against severe clinical disease of SARS-CoV-2 in hamsters. This indicates that the vaccine protects against the SARS-CoV-2 infection (46). According to the CT analysis, Gam-COVID-Vac and Sputnik light vaccine demonstrated good efficacy, safety, and tolerance against COVID-19 infection (194–196, 202, 262, 263). Thus, based on the proven efficacy and safety of these vaccinations, both Gam-COVID-Vac and Sputnik light vaccines received approval for emergency use in Russia and numerous other countries (264).

The approval and emergency usage authorization of several COVID-19 vaccines worldwide have made significant progress in fighting against SARS-CoV-2. However, the emergence of mutant strains, which can re-infect a large number of previously immunized individuals, has renewed concerns about potential vaccine vulnerability. Therefore, a comprehensive analysis of the current development status of vaccines provides a theoretical basis for guiding the design and deployment of vaccines in the future.

## Conclusions

The COVID-19 pandemic brought severe challenges to the global economic burden, medical institutions, and medical infrastructure. It is time-consuming to collaborate with a wide range of expertise to achieve innovative and effective solutions. Vaccines are an important strategy in the management of viral transmission. One of challenges of COVID-19 vaccines is the ADE effect. From the beginning of the COVID-19 vaccine development, scientists and researchers attempted to identify a SARS-CoV-2 protein, which elicited the least ADE effect. Thus far, no ADE effect was observed in the animal studies and human clinical trials. However, the ADE effect requires additional investigation, as it involves both vaccine development and treatment.

In addition, vaccines must be used effectively, and in combination with other evidence-based public health measures, in order to play a decisive role. Furthermore, the efficacy of SARS-CoV-2 novel vaccines must be critically evaluated, with a scientific and rigorous attitude, in order to understand its universality and clinical significance. Considering the nature of SARS-CoV-2, the vaccine may require regular modifications, and other scientific questions regarding novel vaccines will need to be answered, particularly, in terms of vaccine design and improvement of vaccine effectiveness, including antigen screening, administration route, clinical trials, vaccine safety, effectiveness, dose enhancement, optimization of vaccination regimens, strengthening of post-vaccination monitoring, and so on. Timely and coordinated implementation of these post-vaccination tasks will effectively and efficiently bring the pandemic to an end.

## Author Contributions

ZZ and QS prepared the initial drafts of the manuscript and contributed equally to this work. HC was mainly responsible for revising the manuscript. All of the authors approved the final version of the manuscript.

## Funding

The work was supported by the National Natural Science Foundation of China (62005085), and the China Postdoctoral Science Foundation (2021M691093).

## Conflict of Interest

The authors declare that the research was conducted in the absence of any commercial or financial relationships that could be construed as a potential conflict of interest.

## Publisher’s Note

All claims expressed in this article are solely those of the authors and do not necessarily represent those of their affiliated organizations, or those of the publisher, the editors and the reviewers. Any product that may be evaluated in this article, or claim that may be made by its manufacturer, is not guaranteed or endorsed by the publisher.

## References

[1] COVID-19 - China. WHO Disease Outbreak News (2020). Available at: https://www.who.int/emergencies/disease-outbreak-news/item/2020-DON229.

[2] Novel Coronavirus (2019-nCoV). WHO Situation Report-1 (2020). Available at: https://www.who.int/emergencies/diseases/novel-coronavirus-2019/situation-reports.

[3] WHO Technical Guidance. Naming the Coronavirus Disease (COVID-19) and the Virus That Causes It (2020). Available at: https://www.who.int/emergencies/diseases/novel-coronavirus-2019/technical-guidance/naming-the-coronavirus-disease-(covid-2019)-and-the-virus-that-causes-it.

[4] WHO Coronavirus (COVID-19) Dashboard. WHO Global Situation (2021). Available at: https://covid19.who.int/.

[5] Kuehn BM. Most Patients Hospitalized With COVID-19 Have Lasting Symptoms. Jama (2021) 325:1031. doi: 10.1001/jama.2021.2974 33724332

[6] Kupferschmidt K Cohen J. Race to Find COVID-19 Treatments Accelerates. Science (2020) 367:1412–3. doi: 10.1126/science.367.6485.1412 32217705

[7] Krammer F. SARS-CoV-2 Vaccines in Development. Nature (2020) 586:516–27. doi: 10.1038/s41586-020-2798-3 32967006

[8] Finkel Y Mizrahi O Nachshon A Weingartengabbay S Morgenstern D Yahalomronen Y. The Coding Capacity of SARS-CoV-2. Nature (2020) 589:125–30. doi: 10.1038/s41586-020-2739-1 32906143

[9] Lei X Dong X Ma R Wang W Wang J. Activation and Evasion of Type I Interferon Responses by SARS-CoV-2. Nat Commun (2020) 11:3810. doi: 10.1038/s41467-020-17665-9 32733001 PMC7392898

[10] Xia H Cao Z Xie X Zhang X Shi PY. Evasion of Type I Interferon by SARS-CoV-2. Cell Rep (2020) 33:108234. doi: 10.1016/j.celrep.2020.108234 32979938 PMC7501843

[11] Shen Q Li J Zhang Z Guo S Wang Q An X. COVID-19: Systemic Pathology and Its Implications for Therapy. Int J Biol Sci (2022) 18:386–408. doi: 10.7150/ijbs.65911 34975340 PMC8692150

[12] Fan XY Cao DF Kong LF Zhang XZ. Cryo-EM Analysis of the Post-Fusion Structure of the SARS-CoV Spike Glycoprotein. Nat Commun (2020) 11:3618. doi: 10.1038/s41467-020-17371-6 32681106 PMC7367865

[13] Hoffmann M Kleine-Weber H Schroeder S Krüger N Herrler T Erichsen S. SARS-CoV-2 Cell Entry Depends on ACE2 and TMPRSS2 and Is Blocked by a Clinically Proven Protease Inhibitor. Cell (2020) 181:271–80.e8. doi: 10.1016/j.cell.2020.02.052 32142651 PMC7102627

[14] Du L He Y Zhou Y Liu S Zheng BJ Jiang S. The Spike Protein of SARS-CoV–a Target for Vaccine and Therapeutic Development. Nat Rev Microbiol (2009) 7:226–36. doi: 10.1038/nrmicro2090 PMC2750777 19198616

[15] Schoeman D Fielding BC. Coronavirus Envelope Protein: Current Knowledge. Virol J (2019) 16:69. doi: 10.1186/s12985-019-1182-0 31133031 PMC6537279

[16] Dey D Borkotoky S Banerjee M. In Silico Identification of Tretinoin as a SARS-CoV-2 Envelope (E) Protein Ion Channel Inhibitor. Comput Biol Med (2020) 127:104063. doi: 10.1016/j.compbiomed.2020.104063 33126128 PMC7574788

[17] Nieto-Torres J Dediego M Alvarez E Jiménez-Guardeño J Regla-Nava J Llorente M. Subcellular Location and Topology of Severe Acute Respiratory Syndrome Coronavirus Envelope Protein. Virology (2011) 415:69–82. doi: 10.1016/j.virol.2011.03.029 21524776 PMC4726981

[18] Chang CK Hou MH Chang CF Hsiao CD Huang TH. The SARS Coronavirus Nucleocapsid Protein - Forms and Functions. Antiviral Res (2014) 103:39–50. doi: 10.1016/j.antiviral.2013.12.009 24418573 PMC7113676

[19] Cui L Wang H Ji Y Yang J Xu S Huang X. The Nucleocapsid Protein of Coronaviruses Acts as a Viral Suppressor of RNA Silencing in Mammalian Cells. J Virol (2015) 89:9029–43. doi: 10.1128/JVI.01331-15 PMC4524063 26085159

[20] Oliveira SC Magalhes MTQ Homan EJ. Immunoinformatic Analysis of SARS-CoV-2 Nucleocapsid Protein and Identification of COVID-19 Vaccine Targets. Front Immunol (2020) 11:587615. doi: 10.3389/fimmu.2020.587615 33193414 PMC7655779

[21] Moriguchi T Harii N Goto J Harada D Sugawara H Takamino J. A First Case of Meningitis/Encephalitis Associated With SARS-Coronavirus-2. Int J Infect Dis IJID (2020) 94:55–58. doi: 10.1016/j.ijid.2020.03.062 32251791 PMC7195378

[22] Kirchdoerfer RN Ward AB. Structure of the SARS-CoV Nsp12 Polymerase Bound to Nsp7 and Nsp8 Co-Factors. Nat Commun (2019) 10:2342. doi: 10.1038/s41467-019-10280-3 31138817 PMC6538669

[23] Ahmad Abu Turab Naqvi KF. Insights Into SARS-CoV-2 Genome, Structure, Evolution, Pathogenesis and Therapies: Structural Genomics Approach %. Taj Mohammada J Biochim Biophys Acta (BBA) - Mol Basis Dis (2020) 1866:165878. doi: 10.1016/j.bbadis.2020.165878 PMC7293463 32544429

[24] Jia Z Yan L Ren Z Wu L Wang J Guo J. Delicate Structural Coordination of the Severe Acute Respiratory Syndrome Coronavirus Nsp13 Upon ATP Hydrolysis. Nuclc Acids Res (2019) 47:6538–50. doi: 10.1093/nar/gkz409 PMC6614802 31131400

[25] Ferron F Subissi L De Morais ATS Le N Sevajol M Gluais L. Structural and Molecular Basis of Mismatch Correction and Ribavirin Excision From Coronavirus RNA. Proc Natl Acad Sci USA (2018) 115:E162–E71. doi: 10.1073/pnas.1718806115 PMC5777078 29279395

[26] Zhang Y Zhang J Chen Y Luo B Zhang H. The ORF8 Protein of SARS-CoV-2 Mediates Immune Evasion Through Potently Downregulating MHC-I. bioRxiv (2020):2020.2005.2024.111823. doi: 10.1101/2020.05.24.111823

[27] Jiang HW Zhang HN Meng QF Xie J Li Y Chen H. SARS-CoV-2 Orf9b Suppresses Type I Interferon Responses by Targeting TOM70. Cell Mol Immunol (2020) 17:998–1000. doi: 10.1038/s41423-020-0514-8 32728199 PMC7387808

[28] Hassan SS Attrish D Ghosh S Choudhury PP Brufsky AM. Notable Sequence Homology of the ORF10 Protein Introspects the Architecture of SARS-COV-2. Int J Biol Macromol (2020) 181:801–9. doi: 10.1016/j.ijbiomac.2021.03.199 PMC8051021 33862077

[29] Tirado SM Yoon KJ. Antibody-Dependent Enhancement of Virus Infection and Disease. Viral Immunol (2003) 16:69–86. doi: 10.1089/088282403763635465 12725690

[30] Long Q-X Liu B-Z Deng H-J Wu G-C Deng K Chen Y-K. Antibody Responses to SARS-CoV-2 in Patients With COVID-19. Nat Med (2020) 26:845–8. doi: 10.1038/s41591-020-0897-1 32350462

[31] Liu Y Soh WT Kishikawa JI Hirose M Nakayama EE Li S. An Infectivity-Enhancing Site on the SARS-CoV-2 Spike Protein Targeted by Antibodies. Cell (2021) 184:3452–66.e18. doi: 10.1016/j.cell.2021.05.032 34139176 PMC8142859

[32] Dudas G Hong S Potter B Calvignac-Spencer S Niatou-Singa F Tombolomako T. Travel-Driven Emergence and Spread of SARS-CoV-2 Lineage B.1.620 With Multiple VOC-Like Mutations and DeletionsIn Europe. (2021) 97:662–7. doi: 10.1101/2021.05.04.21256637 PMC8486757 34599175

[33] Liu L Wei Q Lin Q Fang J Wang H Kwok H. Anti-Spike IgG Causes Severe Acute Lung Injury by Skewing Macrophage Responses During Acute SARS-CoV Infection. JCI Insight (2019) 4:2379–3708. doi: 10.1172/jci.insight.123158 PMC6478436 30830861

[34] Tan SC Yiap BC. DNA, RNA, and Protein Extraction: The Past and the Present. J Biomed Biotechnol (2009) 2009:574398. doi: 10.1155/2009/574398 20011662 PMC2789530

[35] Iversen PL Bavari S. Inactivated COVID-19 Vaccines to Make a Global Impact. Lancet Infect Dis (2021) 21:746–8. doi: 10.1016/S1473-3099(21)00020-7 PMC7906657 33548196

[36] Rawat K Kumari P Saha L. COVID-19 Vaccine: A Recent Update in Pipeline Vaccines, Their Design and Development Strategies. Eur J Pharmacol (2021) 892:173751. doi: 10.1016/j.ejphar.2020.173751 33245898 PMC7685956

[37] Dai L Gao GF. Viral Targets for Vaccines Against COVID-19. Nat Rev Immunol (2021) 21:73–82. doi: 10.1038/s41577-020-00480-0 33340022 PMC7747004

[38] Saputri DS Li S van Eerden FJ Rozewicki J Xu Z Ismanto HS. Flexible, Functional, and Familiar: Characteristics of SARS-CoV-2 Spike Protein Evolution. Front Microbiol (2020) 11:2112. doi: 10.3389/fmicb.2020.02112 33042039 PMC7527407

[39] Ghorbani M Brooks BR Klauda JB. Exploring Dynamics and Network Analysis of Spike Glycoprotein of SARS-COV-2. Biophys J (2021) 120:2902–13. doi: 10.1016/j.bpj.2021.02.047 PMC7939993 33705760

[40] Corbett KS Edwards DK Leist SR Abiona OM Boyoglu-Barnum S Gillespie RA. SARS-CoV-2 mRNA Vaccine Design Enabled by Prototype Pathogen Preparedness. Nature (2020) 586:567–71. doi: 10.1038/s41586-020-2622-0 PMC7581537 32756549

[41] Heath PT Galiza EP Baxter DN Boffito M Browne D Burns F. Safety and Efficacy of NVX-CoV2373 Covid-19 Vaccine. New Engl J Med (2021) 385:1172–83. doi: 10.1056/NEJMoa2107659 PMC8262625 34192426

[42] Jackson LA Anderson EJ Rouphael NG Roberts PC Makhene M Coler RN. An mRNA Vaccine Against SARS-CoV-2 - Preliminary Report. New Engl J Med (2020) 383:1920–31. doi: 10.1056/NEJMoa2022483 PMC7377258 32663912

[43] Mercado NB Zahn R Wegmann F Loos C Chandrashekar A Yu J. Single-Shot Ad26 Vaccine Protects Against SARS-CoV-2 in Rhesus Macaques. Nature (2020) 586:583–8. doi: 10.1038/s41586-020-2607-z PMC7581548 32731257

[44] Sadoff J Gray G Vandebosch A Cardenas V Shukarev G Grinsztejn B. Safety and Efficacy of Single-Dose Ad26.Cov2.S Vaccine Against Covid-19. New Engl J Med (2021) 384:2187–201. doi: 10.1056/NEJMoa2101544 PMC8220996 33882225

[45] Sadoff J Le Gars M Shukarev G Heerwegh D Truyers C de Groot AM. Interim Results of a Phase 1-2a Trial of Ad26.COV2.S Covid-19 Vaccine. New Engl J Med (2021) 384:1824–35. doi: 10.1056/NEJMoa2034201 PMC7821985 33440088

[46] Tostanoski LH Wegmann F Martinot AJ Loos C McMahan K Mercado NB. Ad26 Vaccine Protects Against SARS-CoV-2 Severe Clinical Disease in Hamsters. Nat Med (2020) 26:1694–700. doi: 10.1038/s41591-020-1070-6 PMC7671939 32884153

[47] Walsh EE Frenck R Falsey AR Kitchin N Absalon J Gurtman A. RNA-Based COVID-19 Vaccine BNT162b2 Selected for a Pivotal Efficacy Study. medRxiv (2020). doi: 10.1101/2020.08.17.20176651

[48] Walsh EE Frenck RW Jr Falsey AR Kitchin N Absalon J Gurtman A. Safety and Immunogenicity of Two RNA-Based Covid-19 Vaccine Candidates. New Engl J Med (2020) 383:2439–50. doi: 10.1056/NEJMoa2027906 PMC7583697 33053279

[49] Awadasseid A Wu Y Tanaka Y Zhang W. Current Advances in the Development of SARS-CoV-2 Vaccines. Int J Biol Sci (2021) 17:8–19. doi: 10.7150/ijbs.52569 33390829 PMC7757035

[50] McBride R van Zyl M Fielding BC. The Coronavirus Nucleocapsid is a Multifunctional Protein. Viruses (2014) 6:2991–3018. doi: 10.3390/v6082991 25105276 PMC4147684

[51] Holmes KV Enjuanes L. Virology. The SARS Coronavirus: A Postgenomic Era. Science (2003) 300:1377–8. doi: 10.1126/science.1086418 12775826

[52] Hu H Huang X Tao L Huang Y Cui BA Wang H. Comparative Analysis of the Immunogenicity of SARS-CoV Nucleocapsid DNA Vaccine Administrated With Different Routes in Mouse Model. Vaccine (2009) 27:1758–63. doi: 10.1016/j.vaccine.2009.01.021 PMC7115532 19186202

[53] Dutta NK Mazumdar K Gordy JT. The Nucleocapsid Protein of SARS-CoV-2: A Target for Vaccine Development. J Virol (2020) 94:e00647–20. doi: 10.1128/JVI.00647-20 PMC7307180 32546606

[54] Yadav R Chaudhary JK Jain N Chaudhary PK Khanra S Dhamija P. Role of Structural and Non-Structural Proteins and Therapeutic Targets of SARS-CoV-2 for COVID-19. Cells (2021) 10:821. doi: 10.3390/cells10040821 33917481 PMC8067447

[55] Fehr AR Perlman S. Coronaviruses: An Overview of Their Replication and Pathogenesis. Methods Mol Biol (2015) 1282:1–23. doi: 10.1007/978-1-4939-2438-7_1 25720466 PMC4369385

[56] Kim YC Dema B Reyes-Sandoval A. COVID-19 Vaccines: Breaking Record Times to First-in-Human Trials. NPJ Vaccines (2020) 5:34. doi: 10.1038/s41541-020-0188-3 32377399 PMC7193619

[57] COVID-19 Vaccines Living Mapping. COVID-19 NMA (2021). Available at: https://covid-nma.com/vaccines/mapping/.

[58] Li Z Xiang T Liang B Deng H Wang H Feng X. Characterization of SARS-CoV-2-Specific Humoral and Cellular Immune Responses Induced by Inactivated COVID-19 Vaccines in a Real-World Setting. Front Immunol (2021) 12:802858. doi: 10.3389/fimmu.2021.802858 35003131 PMC8727357

[59] Al Kaabi N Zhang Y Xia S Yang Y Al Qahtani MM Abdulrazzaq N. Effect of 2 Inactivated SARS-CoV-2 Vaccines on Symptomatic COVID-19 Infection in Adults: A Randomized Clinical Trial. JAMA (2021) 326:35–45. doi: 10.1001/jama.2021.8565 34037666 PMC8156175

[60] Xia S Zhang Y Wang Y Wang H Yang Y Gao GF. Safety and Immunogenicity of an Inactivated SARS-CoV-2 Vaccine, BBIBP-CorV: A Randomised, Double-Blind, Placebo-Controlled, Phase 1/2 Trial. Lancet Infect Dis (2021) 21:39–51. doi: 10.1016/S1473-3099(20)30831-8 33069281 PMC7561304

[61] Wang GL Wang ZY Duan LJ Meng QC Jiang MD Cao J. Susceptibility of Circulating SARS-CoV-2 Variants to Neutralization. New Engl J Med (2021) 384:2354–6. doi: 10.1056/NEJMc2103022 PMC8063885 33822491

[62] Le TT Cramer JP Chen R Mayhew S. Evolution of the COVID-19 Vaccine Development Landscape. Nat Rev Drug Discov (2020) 19:667–8. doi: 10.1038/d41573-020-00151-8 32887942

[63] Wang H Zhang Y Huang B Deng W Quan Y Wang W. Development of an Inactivated Vaccine Candidate, BBIBP-CorV, With Potent Protection Against SARS-CoV-2. Cell (2020) 182:713–21 e9. doi: 10.1016/j.cell.2020.06.008 32778225 PMC7275151

[64] A Phase III Clinical Trial for Inactivated Novel Coronavirus Pneumonia (COVID-19) Vaccine (Vero Cells). ChiCTR (2020). Available at: http://www.chictr.org.cn/showproj.aspx?proj=56651.

[65] A Phase III Clinical Trial for Inactivated Novel Coronavirus Pneumonia (COVID-19) Vaccine (Vero Cells). ChiCTR (2021). Available at: http://www.chictr.org.cn/showproj.aspx?proj=62581.

[66] Comprehensive Evaluations of Dynamic Changes in the Immune System of Healthy Volunteers After Vaccination With Inactivated Novel Coronavirus Pneumonia (COVID-19) Vaccine (Vero Cells). ChiCTR (2021). Available at: http://www.chictr.org.cn/showproj.aspx?proj=122756.

[67] Canceled by the Investigator Evaluation of Immunogenicity and Safety of Combined Immunization of COVIV and PPV23 / Iiv4. ChiCTR (2021). Available at: http://www.chictr.org.cn/showproj.aspx?proj=126277.

[68] Evaluation of Immunogenicity and Safety of Combined Immunization of COVIV and PPV23 / IIV4. ChiCTR (2021). Available at: http://www.chictr.org.cn/showproj.aspx?proj=126385.

[69] A Study to Evaluate The Efficacy, Safety and Immunogenicity of Inactivated SARS-CoV-2 Vaccines (Vero Cell) in Healthy Population Aged 18 Years Old and Above (COVID-19). NIH (2020). Available at: https://clinicaltrials.gov/ct2/show/NCT04510207.

[70] Efficacy, Safety and Immunogenicity of Inactivated SARS-CoV-2 Vaccines (Vero Cell) to Prevent COVID-19 in Healthy Adult Population In Peru Healthy Adult Population In Peru (Covid-Peru). NIH (2020). Available at: https://clinicaltrials.gov/ct2/show/NCT04612972.

[71] The Efficacy, Safety and Immunogenicity Study of Inactivated SARS-CoV-2 Vaccine for Preventing Against COVID-19. NIH (2020). Available at: https://clinicaltrials.gov/ct2/show/NCT04659239.

[72] Zhang Y Zeng G Pan H Li C Hu Y Chu K. Safety, Tolerability, and Immunogenicity of an Inactivated SARS-CoV-2 Vaccine in Healthy Adults Aged 18-59 Years: A Randomised, Double-Blind, Placebo-Controlled, Phase 1/2 Clinical Trial. Lancet Infect Dis (2021) 21:181–92. doi: 10.1016/S1473-3099(20)30843-4 PMC7832443 33217362

[73] Bueno SM Abarca K Gonzalez PA Galvez NMS Soto JA Duarte LF. Safety and Immunogenicity of an Inactivated SARS-CoV-2 Vaccine in a Subgroup of Healthy Adults in Chile. Clin Infect Dis (2021). doi: 10.1093/cid/ciab823 PMC9402626 34537835

[74] Fadlyana E Rusmil K Tarigan R Rahmadi AR Prodjosoewojo S Sofiatin Y. A Phase III, Observer-Blind, Randomized, Placebo-Controlled Study of the Efficacy, Safety, and Immunogenicity of SARS-CoV-2 Inactivated Vaccine in Healthy Adults Aged 18-59 Years: An Interim Analysis in Indonesia. Vaccine (2021) 39:6520–8. doi: 10.1016/j.vaccine.2021.09.052 PMC8461222 34620531

[75] Tanriover MD Doganay HL Akova M Guner HR Azap A Akhan S. Efficacy and Safety of an Inactivated Whole-Virion SARS-CoV-2 Vaccine (CoronaVac): Interim Results of a Double-Blind, Randomised, Placebo-Controlled, Phase 3 Trial in Turkey. Lancet (2021) 398:213–22. doi: 10.1016/S0140-6736(21)01429-X PMC8266301 34246358

[76] Chen Y Shen H Huang R Tong X Wu C. Serum Neutralising Activity Against SARS-CoV-2 Variants Elicited by CoronaVac. Lancet Infect Dis (2021) 21:1071–2. doi: 10.1016/S1473-3099(21)00287-5 PMC8159188 34051887

[77] Clinical Trial of Efficacy and Safety of Sinovac’s Adsorbed COVID-19 (Inactivated) Vaccine in Healthcare Professionals (PROFISCOV). NIH (2020). Available at: https://clinicaltrials.gov/ct2/show/NCT04456595.

[78] Clinical Trial For SARS-CoV-2 Vaccine (COVID-19). NIH (2020). Available at: https://clinicaltrials.gov/ct2/show/NCT04582344.

[79] Efficacy, Safety, and Immunogenicity of Two Vaccination Schedules of an Inactivated Vaccine Against COVID-19 in Adults (CoronaVac3CL). NIH (2020). Available at: https://clinicaltrials.gov/ct2/show/NCT04651790.

[80] An Effectiveness Study of the Sinovac’s Adsorbed COVID-19 (Inactivated) Vaccine (Projeto S). NIH (2021). Available at: https://clinicaltrials.gov/ct2/show/NCT04747821.

[81] A Study to Assess the Safety and Immunogenicity of the Coronavac Vaccine Against COVID-19. NIH (2021). Available at: https://clinicaltrials.gov/ct2/show/NCT04756830.

[82] Antibody Response to COVID-19 Vaccines in Liver Disease Patients. NIH (2021). Available at: https://clinicaltrials.gov/ct2/show/NCT04775069.

[83] Effectiveness of the Adsorbed Vaccine COVID-19 (Coronavac) Among Education and Public Safety Workers With Risk Factors for Severity (COVACMANAUS). NIH (2021). Available at: https://clinicaltrials.gov/ct2/show/NCT04789356.

[84] Safety of an Inactivated SARS-CoV-2 Vaccine for Prevention of COVID-19 in Adults. NIH (2021). Available at: https://clinicaltrials.gov/ct2/show/NCT04911790.

[85] Efficacy, Immunogenicity, and Safety of the Inactivated COVID-19 Vaccine (TURKOVAC) Versus the CoronaVac Vaccine. NIH (2021). Available at: https://clinicaltrials.gov/ct2/show/NCT04942405.

[86] Immunogenicity and Safety of an Inactivated COVID-19 Vaccine. NIH (2021). Available at: https://clinicaltrials.gov/ct2/show/NCT04953325.

[87] Gao Q Bao L Mao H Wang L Xu K Yang M. Development of an Inactivated Vaccine Candidate for SARS-CoV-2. Science (2020) 369:77–81. doi: 10.1126/science.abc1932 32376603 PMC7202686

[88] Lu L Mok BW Chen LL Chan JM Tsang OT Lam BH. Neutralization of SARS-CoV-2 Omicron Variant by Sera From BNT162b2 or Coronavac Vaccine Recipients. Clin Infect Dis (2021). doi: 10.1101/2021.12.13.21267668 PMC8754807 34915551

[89] Sapkal GN Yadav PD Ella R Deshpande GR Sahay RR Gupta N. Inactivated COVID-19 Vaccine BBV152/COVAXIN Effectively Neutralizes Recently Emerged B.1.1.7 Variant of SARS-CoV-2. J Travel Med (2021) 28:taab051. 33772577 10.1093/jtm/taab051 PMC8083765

[90] Sapkal G Yadav PD Ella R Abraham P Patil DY Gupta N. Neutralization of VUI B.1.1.28 P2 Variant With Sera of COVID-19 Recovered Cases and Recipients of Covaxin an Inactivated COVID-19 Vaccine. J Travel Med (2021) 28:taab077. doi: 10.1093/jtm/taab077 34002240 PMC8194512

[91] COVAXIN(TM) (BBV152) Booster Shown to Neutralize Both Omicron and Delta Variants of SARS-CoV-2. Stockhouse News (2022). Available at: https://stockhouse.com/news/press-releases/2022/01/12/covaxin-tm-bbv152-booster-shown-to-neutralize-both-omicron-and-delta-variants-of.

[92] Study: Covaxin COVID-19 Booster Neutralizes Delta and Omicron Variants. Pharmacytimes (2022). Available at: https://www.pharmacytimes.com/view/study-covaxin-covid-19-booster-neutralizes-delta-and-omicron-variants.

[93] Ella R Reddy S Blackwelder W Potdar V Yadav P Sarangi V. Efficacy, Safety, and Lot-to-Lot Immunogenicity of an Inactivated SARS-CoV-2 Vaccine (BBV152): Interim Results of a Randomised, Double-Blind, Controlled, Phase 3 Trial. Lancet (2021) 398:2173–84. doi: 10.1101/2021.06.30.21259439 PMC8584828 34774196

[94] An Efficacy and Safety Clinical Trial of an Investigational COVID-19 Vaccine (BBV152) in Adult Volunteers. NIH (2020). Available at: https://clinicaltrials.gov/ct2/show/NCT04641481.

[95] Comparison of Reactogenicity and Immunogenicity of Heterologous Prime-Boost and Heterologous Boost of ChAdOx1 Ncov-19 (Covishield), BBV 152 (Covaxin), and Other COVID Vaccines With Homologous Administration of Covishield and Covaxin CTRI (2021). Available at: http://www.ctri.nic.in/Clinicaltrials/pmaindet2.php?trialid=59405.

[96] Ariamanesh M Porouhan P PeyroShabany B Fazilat-Panah D Dehghani M Nabavifard M. Immunogenicity and Safety of the Inactivated SARS-CoV-2 Vaccine (BBIBP-CorV) in Patients With Malignancy. Cancer Invest (2021) 40:26–34. doi: 10.1101/2021.09.02.21262760 34634986 PMC8567287

[97] Jeewandara C Aberathna IS Pushpakumara PD Kamaladasa A Guruge D Wijesinghe A. Persistence of Antibody and T Cell Responses to the Sinopharm/BBIBP-CorV Vaccine in Sri Lankan Individuals. medRxiv (2021). doi: 10.1101/2021.10.14.21265030

[98] Clinical Trial to Evaluate the Efficacy, Immunogenicity and Safety of the Inactivated SARS-CoV-2 Vaccine (COVID-19). NIH (2020). Available at: https://clinicaltrials.gov/ct2/show/NCT04560881.

[99] Immuno-Bridging Study of Inactivated SARS-CoV-2 Vaccine in Healthy Population Aged 3-17 vs Aged 18 Years Old and Above (COVID-19). NIH (2021). Available at: https://clinicaltrials.gov/ct2/show/NCT04917523.

[100] Efficacy, Immunogenicity and Safety of BBIBP-CorV Vaccine Against Severe Acute Respiratory Syndrome Coronavirus 2 (SARS-CoV-2) Infection. (ECOVA-01). NIH (2021). Available at: https://clinicaltrials.gov/ct2/show/NCT04984408.

[101] Immunogenicity and Safety After Vaccinated With BBIBP-CorV (COVID-19 Vaccine) in Thai Pregnant Women. TCTR (2021). Available at: https://www.thaiclinicaltrials.org/show/TCTR20210923013.

[102] A Immuno-Bridging and Immunization Schedules Study of COVID-19 Vaccine (Vero Cell), Inactivated (COVID-19). NIH (2021). Available at: https://clinicaltrials.gov/ct2/show/NCT04863638.

[103] Safety and Immunogenicity of Full or Half Dose of a COVID-19 Booster Vaccine After Completion of Two-Dose Inactivated and Viral Vector Vaccines. TCTR (2021). Available at: https://www.thaiclinicaltrials.org/show/TCTR20210910002.

[104] Evaluation of Immunogenicity and Safety of Combined Immunization of COVAX (Produced in Wuhan) and PPV23 / IIV4. NIH (2021). Available at: https://clinicaltrials.gov/ct2/show/NCT05079152.

[105] Immunogenicity and Safety of a Third Dose and Immune Persistence of BBIBP-Corv Vaccine in People With HIV Infected. NIH (2021). Available at: https://clinicaltrials.gov/ct2/show/NCT05105295.

[106] Immunogenicity and Safety of a Third Dose and Immune Persistence of BBIBP-Corv Vaccine in Elderly People With Chronic Bronchitis and COPD. NIH (2021). Available at: https://clinicaltrials.gov/ct2/show/NCT05104216.

[107] Immunogenicity, Efficacy and Safety of QazCovid-In® COVID-19 Vaccine. NIH (2021). Available at: https://www.clinicaltrials.gov/ct2/show/NCT04691908?id=NCT04639466+OR+NCT04659941+OR+NCT04691947+OR+NCT04651790+OR+NCT04659239+OR+NCT04648800+OR+NCT04691908+OR+NCT04656613+OR+NCT04672395+OR+NCT04673149+OR+NCT04671017+OR+NCT04685603+OR+NCT04664309+OR+NCT04686773+OR+NCT04681092+OR+NCT04662697+OR+NCT04652102+OR+NCT04665258+OR+NCT04649021+OR+NCT04686409+OR+NCT04690387+OR+NCT04666012+OR+NCT04649151+OR+NCT04655625+OR+NCT04684446+OR+NCT04668339+OR+NCT04683224+OR+NCT04674189+OR+NCT04690816+OR+NCT04679909&draw=2&rank=2&load=cart.

[108] QazCovid-In. Economic Research Institute (2020). Available at: https://economy.kz/en/Novosti_ekonomiki_Kazahstana/id=1588.

[109] Study To Compare The Immunogenicity Against COVID-19, Of VLA2001 Vaccine To AZD1222 Vaccine (COV-COMPARE). NIH (2021). Available at: https://clinicaltrials.gov/ct2/show/NCT04864561.

[110] Immunogenicity of VLA2101 Compared to VLA2001. NIH (2021). Available at: https://clinicaltrials.gov/ct2/show/NCT04956224.

[111] Polack FP Thomas SJ Kitchin N Absalon J Gurtman A Lockhart S. Safety and Efficacy of the BNT162b2 mRNA Covid-19 Vaccine. New Engl J Med (2020) 383:2603–15. doi: 10.1056/NEJMoa2034577 PMC7745181 33301246

[112] Dickerman BA Gerlovin H Madenci AL Kurgansky KE Ferolito BR Figueroa Muniz MJ. Comparative Effectiveness of BNT162b2 and mRNA-1273 Vaccines in US. Veterans. New Engl J Med (2022) 386:105–15. doi: 10.1056/NEJMoa2115463 PMC8693691 34942066

[113] Lamb YN. BNT162b2 mRNA COVID-19 Vaccine: First Approval. Drugs (2021) 81:495–501. doi: 10.1007/s40265-021-01480-7 33683637 PMC7938284

[114] Sahin U Muik A Vogler I Derhovanessian E Kranz LM Vormehr M. BNT162b2 Vaccine Induces Neutralizing Antibodies and Poly-Specific T Cells in Humans. Nature (2021) 595:572–7. doi: 10.1038/s41586-021-03653-6 34044428

[115] Frenck RW Jr Klein NP Kitchin N Gurtman A Absalon J Lockhart S. Safety, Immunogenicity, and Efficacy of the BNT162b2 Covid-19 Vaccine in Adolescents. New Engl J Med (2021) 385:239–50. doi: 10.1056/NEJMoa2107456 PMC8174030 34043894

[116] A Phase 3 Study to Evaluate the Safety, Tolerability, and Immunogenicity of Multiple Production Lots and Dose Levels of BNT162b2 RNA-Based COVID-19 Vaccines Against COVID-19 in Healthy Participants. NIH (2021). Available at: https://clinicaltrials.gov/ct2/show/NCT04713553.

[117] Study to Evaluate the Safety, Tolerability, and Immunogenicity of SARS CoV-2 RNA Vaccine Candidate (BNT162b2) Against COVID-19 in Healthy Pregnant Women 18 Years of Age and Older. NIH (2021). Available at: https://clinicaltrials.gov/ct2/show/NCT04754594.

[118] Covid-19 Vaccination in Adolescents and Children (COVAC). NIH (2021). Available at: https://clinicaltrials.gov/ct2/show/NCT04800133.

[119] A Study to Evaluate Safety, Tolerability, & Immunogenicity of Multiple Formulations of BNT162b2 Against COVID-19 in Healthy Adults. NIH (2021). Available at: https://clinicaltrials.gov/ct2/show/NCT04816669.

[120] Study to Evaluate the Safety and Efficacy of a Booster Dose of BNT162b2 Against COVID-19 in Participants ≥16 Years of Age. NIH (2021). Available at: https://clinicaltrials.gov/ct2/show/NCT04955626.

[121] COVID-19: Safety and Immunogenicity of a Reduced Dose of the BioNTech/Pfizer BNT162b2 Vaccine (REDU-VAC). NIH (2021). Available at: https://clinicaltrials.gov/ct2/show/NCT04852861.

[122] Impact of the Immune System on Response to Anti-Coronavirus Disease 19 (COVID-19) Vaccine in Allogeneic Stem Cell Recipients (Covid Vaccin Allo). NIH (2021). Available at: https://clinicaltrials.gov/ct2/show/NCT04951323.

[123] Baden LR El Sahly HM Essink B Kotloff K Frey S Novak R. Efficacy and Safety of the mRNA-1273 SARS-CoV-2 Vaccine. New Engl J Med (2021) 384:403–16. doi: 10.1056/NEJMoa2035389 PMC7787219 33378609

[124] El Sahly HM Baden LR Essink B Doblecki-Lewis S Martin JM Anderson EJ. Efficacy of the mRNA-1273 SARS-CoV-2 Vaccine at Completion of Blinded Phase. New Engl J Med (2021) 385:1774–85. doi: 10.1056/NEJMoa2113017 PMC8482810 34551225

[125] Chu L McPhee R Huang W Bennett H Pajon R Nestorova B. A Preliminary Report of a Randomized Controlled Phase 2 Trial of the Safety and Immunogenicity of mRNA-1273 SARS-CoV-2 Vaccine. Vaccine (2021) 39:2791–9. doi: 10.1016/j.vaccine.2021.02.007 PMC7871769 33707061

[126] Wu K Choi A Koch M Ma L Hill A Nunna N. Preliminary Analysis of Safety and Immunogenicity of a SARS-CoV-2 Variant Vaccine Booster. medRxiv (2021) 2021.05.05.21256716. doi: 10.1101/2021.05.05.21256716

[127] A Study to Evaluate Efficacy, Safety, and Immunogenicity of mRNA-1273 Vaccine in Adults Aged 18 Years and Older to Prevent COVID-19. NIH (2020). Available at: https://clinicaltrials.gov/ct2/show/NCT04470427.

[128] Immunocompromised Swiss Cohorts Based Trial Platform (COVERALL). NIH (2021). Available at: https://clinicaltrials.gov/ct2/show/NCT04805125.

[129] COVID-19 Vaccine in Immunosuppressed Adults With Autoimmune Diseases (COVIAAD). NIH (2021). Available at: https://clinicaltrials.gov/ct2/show/NCT04806113.

[130] A Study of SARS CoV-2 Infection and Potential Transmission in Individuals Immunized With Moderna COVID-19 Vaccine (CoVPN 3006). NIH (2021). Available at: https://clinicaltrials.gov/ct2/show/NCT04811664.

[131] A Study to Evaluate Safety and Immunogenicity of mRNA-1273 Vaccine to Prevent COVID-19 in Adult Organ Transplant Recipients and in Healthy Adult Participants. NIH (2021). Available at: https://clinicaltrials.gov/ct2/show/NCT04860297.

[132] Third Dose of Moderna COVID-19 Vaccine in Transplant Recipients. NIH (2021). Available at: https://clinicaltrials.gov/ct2/show/NCT04885907.

[133] SARS-CoV-2 Immune Responses After COVID-19 Therapy and Subsequent Vaccine. NIH (2021). Available at: https://clinicaltrials.gov/ct2/show/NCT04952402.

[134] Hein S Herrlein ML Mhedhbi I Bender D Haberger V Benz N. Analysis of BNT162b2- and CVnCoV-Elicited Sera and of Convalescent Sera Toward SARS-CoV-2 Viruses. Allergy (2021). doi: 10.1111/all.15189 34820854

[135] Yadav PD Kumar S. Global Emergence of SARS-CoV-2 Variants: New Foresight Needed for Improved Vaccine Efficacy. Lancet Infect Dis (2022) 22:298–9. doi: 10.1016/S1473-3099(21)00687-3 PMC8610424 34826380

[136] Kremsner PG Mann P Kroidl A Leroux-Roels I Schindler C Gabor JJ. Safety and Immunogenicity of an mRNA-Lipid Nanoparticle Vaccine Candidate Against SARS-CoV-2 : A Phase 1 Randomized Clinical Trial. Wiener Klinische Wochenschrift (2021) 133:931–41. doi: 10.1007/s00508-021-01922-y PMC8354521 34378087

[137] A Study to Evaluate the Safety and Immunogenicity of Vaccine CVnCoV in Healthy Adults in Germany for COVID-19. NIH (2020). Available at: https://clinicaltrials.gov/ct2/show/NCT04674189.

[138] A Study to Evaluate the Immunogenicity and Safety of the SARS-CoV-2 mRNA Vaccine CVnCoV in Elderly Adults Compared to Younger Adults for COVID-19. NIH (2021). Available at: https://clinicaltrials.gov/ct2/show/NCT04838847.

[139] A Study to Evaluate Safety, Reactogenicity and Immunogenicity of the SARS-CoV-2 mRNA Vaccine CVnCoV in Adults With Co-Morbidities for COVID-19. NIH (2021). Available at: https://clinicaltrials.gov/ct2/show/NCT04860258?term=NCT04860258&draw=2&rank=1.

[140] Zhang NN Li XF Deng YQ Zhao H Huang YJ Yang G. A Thermostable mRNA Vaccine Against COVID-19. Cell (2020) 182:1271–83 e16. doi: 10.1016/j.cell.2020.07.024 32795413 PMC7377714

[141] A Phase III Clinical Study of a SARS-CoV-2 Messenger Ribonucleic Acid (mRNA) Vaccine Candidate Against COVID-19 in Population Aged 18 Years and Above. NIH (2021). Available at: https://clinicaltrials.gov/ct2/show/NCT04847102.

[142] Momin T Kansagra K Patel H Sharma S Sharma B Patel J. Safety and Immunogenicity of a DNA SARS-CoV-2 Vaccine (ZyCoV-D): Results of an Open-Label, non-Randomized Phase I Part of Phase I/II Clinical Study by Intradermal Route in Healthy Subjects in India. EClinicalMedicine (2021) 38:101020. doi: 10.1016/j.eclinm.2021.101020 34308319 PMC8285262

[143] A Phase III, Randomized, Multi-Centre, Double Blind, Placebo Controlled, Study to Evaluate Efficacy, Safety and Immunogenicity of Novel Corona Virus -2019-Ncov Vaccine Candidate of M/s Cadila Healthcare Limited. CTRI (2021). Available at: http://www.ctri.nic.in/Clinicaltrials/pmaindet2.php?trialid=51254.

[144] Zydus Cadila has Applied to the Drug Controller General of India for Emergency Use Authorisation of its Plasmid DNA Vaccine, ZyCoV-D. Pharmaceutical Technology (2021). Available at: https://www.pharmaceutical-technology.com/features/worlds-first-dna-covid-19-vaccine/.

[145] Tebas P Yang S Boyer JD Reuschel EL Patel A Christensen-Quick A. Safety and Immunogenicity of INO-4800 DNA Vaccine Against SARS-CoV-2: A Preliminary Report of an Open-Label, Phase 1 Clinical Trial. EClinicalMedicine (2021) 31:100689. doi: 10.1016/j.eclinm.2020.100689 33392485 PMC7759123

[146] Mammen MP Tebas P Agnes J Giffear M Kraynyak KA Blackwood E. Safety and Immunogenicity of INO-4800 DNA Vaccine Against SARS-CoV-2: A Preliminary Report of a Randomized, Blinded, Placebo-Controlled, Phase 2 Clinical Trial in Adults at High Risk of Viral Exposure. medRxiv (2021) 2021:05.07.21256652. doi: 10.1101/2021.05.07.21256652

[147] Safety, Immunogenicity, and Efficacy of INO-4800 for COVID-19 in Adults at High Risk of SARS-CoV-2 Exposure. Pan Afr Clin Trials Registry 12:669339–669339 (2021). doi: 10.3389/fimmu.2021.669339

[148] Yang S Li Y Dai L Wang J He P Li C. Safety and Immunogenicity of a Recombinant Tandem-Repeat Dimeric RBD-Based Protein Subunit Vaccine (ZF2001) Against COVID-19 in Adults: Two Randomised, Double-Blind, Placebo-Controlled, Phase 1 and 2 Trials. Lancet Infect Dis (2021) 21:1107–19. doi: 10.1016/S1473-3099(21)00127-4 PMC7990482 33773111

[149] Zhao X Zheng A Li D Zhang R Sun H Wang Q. Neutralization of Recombinant RBD-Subunit Vaccine ZF2001-Elicited Antisera to SARS-CoV-2 Variants Including Delta. BioRxiv Preprint Server Biol (2021) 2021.07.15.452504. doi: 10.1101/2021.07.15.452504

[150] Zhao X Zheng A Li D Zhang R Sun H Wang Q. Neutralisation of ZF2001-Elicited Antisera to SARS-CoV-2 Variants. Lancet Microbe (2021) 2:e494. doi: 10.1016/S2666-5247(21)00217-2 34458880 PMC8378832

[151] Liu H Zhou C An J Song Y Yu P Li J. Development of Recombinant COVID-19 Vaccine Based on CHO-Produced, Prefusion Spike Trimer and Alum/CpG Adjuvants. Vaccine (2021) 39:7001–11. doi: 10.1016/j.vaccine.2021.10.066 PMC8556577 34750014

[152] A Phase III Clinical Trial to Determine the Safety and Efficacy of ZF2001 for Prevention of COVID-19. NIH (2021). Available at: https://clinicaltrials.gov/ct2/show/NCT04646590.

[153] Clinical Trials of the Consistency and Non-Inferiority Bridging Between Batches of Recombinant New Coronavirus Vaccine (CHO Cells). NIH (2021). Available at: https://clinicaltrials.gov/ct2/show/NCT05091411.

[154] A Randomized, Double-Blind, Placebo-Controlled Phase III Clinical Trial of the Effectiveness and Safety of Inoculation of Recombinant New Coronavirus Vaccine (CHO Cells) in the Prevention of COVID-19 in People 18 Years and Older. ChiCTR (2020). Available at: http://www.chictr.org.cn/showproj.aspx?proj=64718.

[155] A Global Phase III Clinical Trial of Recombinant COVID- 19 Vaccine (Sf9 Cells). NIH (2021). Available at: https://clinicaltrials.gov/ct2/show/NCT04887207.

[156] A Global Phase III Clinical Trial of Recombinant COVID- 19 Vaccine (Sf9 Cells). NIH (2021). Available at: https://clinicaltrials.gov/ct2/show/NCT04904471.

[157] A Study Looking at the Effectiveness, Immune Response, and Safety of a COVID-19 Vaccine in Adults in the United Kingdom. NIH (2020). Available at: https://clinicaltrials.gov/ct2/show/NCT04583995.

[158] A Study to Evaluate the Efficacy, Immune Response, and Safety of a COVID-19 Vaccine in Adults ≥ 18 Years With a Pediatric Expansion in Adolescents (12 to < 18 Years) at Risk for SARS-CoV-2. NIH (2020). Available at: https://clinicaltrials.gov/ct2/show/NCT04611802.

[159] Keech C Albert G Cho I Robertson A Reed P Neal S. Phase 1-2 Trial of a SARS-CoV-2 Recombinant Spike Protein Nanoparticle Vaccine. N Engl J Med (2020) 383:2320–32. doi: 10.1056/NEJMoa2026920 PMC7494251 32877576

[160] Toback S Galiza E Cosgrove C Galloway J Goodman AL Swift PA. Safety, Immunogenicity, and Efficacy of a COVID-19 Vaccine (NVX-CoV2373) Co-Administered With Seasonal Influenza Vaccines: An Exploratory Substudy of a Randomised, Observer-Blinded, Placebo-Controlled, Phase 3 Trial. Lancet Respir Med (2021) 10:167–79. doi: 10.1101/2021.06.09.21258556 PMC8598212 34800364

[161] Study to Evaluate the Safety, Immunogenicity, and Efficacy of Nanocovax Vaccine Against COVID-19. NIH (2021). Available at: https://clinicaltrials.gov/ct2/show/NCT04922788.

[162] A Study to Evaluate Immunogenicity and Safety of MVC-COV1901 Compared With AZD1222 Against COVID-19 in Adults. NIH (2021). Available at: https://clinicaltrials.gov/ct2/show/NCT05011526.

[163] A Heterologous 3rd Boost of MVC-COV1901 to Evaluate Immunogenicity and Safety in Adults With 2 Doses of ChAdOx1-Ncov-19. NIH (2021). Available at: https://clinicaltrials.gov/ct2/show/NCT05097053.

[164] Hsieh SM Liu WD Huang YS Lin YJ Hsieh EF Lian WC. Safety and Immunogenicity of a Recombinant Stabilized Prefusion SARS-CoV-2 Spike Protein Vaccine (MVC-COV1901) Adjuvanted With CpG 1018 and Aluminum Hydroxide in Healthy Adults: A Phase 1, Dose-Escalation Study. EClinicalMedicine (2021) 38:100989. doi: 10.1016/j.eclinm.2021.100989 34222848 PMC8233066

[165] Hsieh SM Liu MC Chen YH Lee WS Hwang SJ Cheng SH. Safety and Immunogenicity of CpG 1018 and Aluminium Hydroxide-Adjuvanted SARS-CoV-2 S-2P Protein Vaccine MVC-COV1901: Interim Results of a Large-Scale, Double-Blind, Randomised, Placebo-Controlled Phase 2 Trial in Taiwan. Lancet Respir Med (2021) 9:1396–406. doi: 10.1016/S2213-2600(21)00402-1 PMC8514195 34655522

[166] Study of the Tolerability, Safety, Immunogenicity and Preventive Efficacy of the EpiVacCorona Vaccine for the Prevention of COVID-19. NIH (2021). Available at: https://clinicaltrials.gov/ct2/show/NCT04780035.

[167] Phase III, Multicenter, Randomized, Double-Blind, Placebo-Controlled Clinical Trial for the Evaluation in Adults of the Efficacy, Safety and Immunogenicity of the Vaccine Candidate CIGB-66 Against SARS-CoV-2. (COVID-19). RPCEC (2021). Available at: https://rpcec.sld.cu/trials/RPCEC00000359-En.

[168] Comparison of the Safety and Efficacy of Razi SARS-CoV-2 Recombinant Spike Protein (Razi Cov Pars) and Sinopharm Vaccines in Adults Aged 18 and Over, a Phase III Randomised, Double Blind, non-Inferiority Clinical Trial. IRCT (2021). Available at: https://en.irct.ir/trial/58143.

[169] Efficacy, Safety, and Immunogenicity of Soberana Recombinant Vaccine (Product of Finlay Institute) Based on RBD Protein Subunit of Sars-Cov-2 in a 2-Dose Regimen With and Without a Booster Dose: A Double-Blind, Randomized, Placebo-Controlled Phase III Clinical Trial in the Iranian Population of 18-80 Years. IRCT (2021). Available at: https://en.irct.ir/trial/54833.

[170] Phase III Clinical Trial, Multicenter, Adaptive, Parallel-Group, Randomized, Placebo-Controlled, Double-Blind Study to Evaluate the Efficacy, Safety and Immunogenicity of Vaccination Against SARS-CoV-2 With 2 Doses of FINLAY-FR-2 and a Heterologous Scheme With 2 Doses of FINLAY-FR-2 and a Booster Dose With FINLAY-FR-1a (COVID-19). RPCEC (2021). Available at: https://rpcec.sld.cu/trials/RPCEC00000354-En.

[171] Ramasamy MN Minassian AM Ewer KJ Flaxman AL Folegatti PM Owens DR. Safety and Immunogenicity of ChAdOx1 Ncov-19 Vaccine Administered in a Prime-Boost Regimen in Young and Old Adults (COV002): A Single-Blind, Randomised, Controlled, Phase 2/3 Trial. Lancet (2021) 396:1979–93. doi: 10.1016/S0140-6736(20)32466-1 PMC7674972 33220855

[172] Phase III Double-Blind, Placebo-Controlled Study of AZD1222 for the Prevention of COVID-19 in Adults. NIH (2020). Available at: https://clinicaltrials.gov/ct2/show/NCT04516746.

[173] A Study of a Candidate COVID-19 Vaccine (COV003). NIH (2020). Available at: https://clinicaltrials.gov/ct2/show/NCT04536051.

[174] AZD1222 Vaccine for the Prevention of COVID-19. NIH (2020). Available at: https://clinicaltrials.gov/ct2/show/NCT04540393.

[175] National Cohort Study of Effectiveness and Safety of SARS-CoV-2/COVID-19 Vaccines (ENFORCE) (ENFORCE). NIH (2020). Available at: https://clinicaltrials.gov/ct2/show/NCT04760132.

[176] Emary KRW Golubchik T Aley PK Ariani CV Angus B Bibi S. Efficacy of ChAdOx1 Ncov-19 (AZD1222) Vaccine Against SARS-CoV-2 Variant of Concern 202012/01 (B.1.1.7): An Exploratory Analysis of a Randomised Controlled Trial. Lancet (2021) 397:1351–62. doi: 10.1016/S0140-6736(21)00628-0 PMC8009612 33798499

[177] Madhi SA Baillie V Cutland CL Voysey M Koen AL Fairlie L. Efficacy of the ChAdOx1 Ncov-19 Covid-19 Vaccine Against the B1.351 Variant. New Engl J Med (2021) 384:1885–98. doi: 10.1056/NEJMoa2102214 PMC7993410 33725432

[178] Voysey M Clemens SAC Madhi SA Weckx LY Folegatti PM Aley PK. Safety and Efficacy of the ChAdOx1 Ncov-19 Vaccine (AZD1222) Against SARS-CoV-2: An Interim Analysis of Four Randomised Controlled Trials in Brazil, South Africa, and the UK. Lancet (2021) 397:99–111. doi: 10.1016/S0140-6736(20)32661-1 33306989 PMC7723445

[179] Voysey M Costa Clemens SA Madhi SA Weckx LY Folegatti PM Aley PK. Single-Dose Administration and the Influence of the Timing of the Booster Dose on Immunogenicity and Efficacy of ChAdOx1 Ncov-19 (AZD1222) Vaccine: A Pooled Analysis of Four Randomised Trials. Lancet (2021) 397:881–91. doi: 10.1016/S0140-6736(21)00432-3 PMC7894131 33617777

[180] Wu S Zhong G Zhang J Shuai L Zhang Z Wen Z. A Single Dose of an Adenovirus-Vectored Vaccine Provides Protection Against SARS-CoV-2 Challenge. Nat Commun (2020) 11:4081. doi: 10.1038/s41467-020-17972-1 32796842 PMC7427994

[181] Wu S Huang J Zhang Z Wu J Zhang J Hu H. Safety, Tolerability, and Immunogenicity of an Aerosolised Adenovirus Type-5 Vector-Based COVID-19 Vaccine (Ad5-Ncov) in Adults: Preliminary Report of an Open-Label and Randomised Phase 1 Clinical Trial. Lancet Infect Dis (2021) 21:1654–64. doi: 10.1016/S1473-3099(21)00396-0 PMC8313090 34324836

[182] Global Phase III Trial of Recombinant Novel Coronavirus Vaccine (Adenovirus Type 5 Vector) (Ad5-Ncov) in Adults 18 Years of Age and Older. ChiCTR (2021). Available at: http://www.chictr.org.cn/showproj.aspx?proj=65034.

[183] Phase III Trial of A COVID-19 Vaccine of Adenovirus Vector in Adults 18 Years Old and Above. NIH (2020). Available at: https://clinicaltrials.gov/ct2/show/NCT04526990.

[184] Clinical Trial of Recombinant Novel Coronavirus Vaccine (Adenovirus Type 5 Vector) Against COVID-19. NIH (2020). Available at: https://clinicaltrials.gov/ct2/show/NCT04540419.

[185] Study on Sequential Immunization of Inactivated SARS-CoV-2 Vaccine and Recombinant SARS-CoV-2 Vaccine (Ad5 Vector). NIH (2021). Available at: https://clinicaltrials.gov/ct2/show/NCT04892459.

[186] Study on Sequential Immunization of Inactivated COVID-19 Vaccine and Recombinant COVID-19 Vaccine (Ad5 Vector) in Elderly Adults. NIH (2021). Available at: https://clinicaltrials.gov/ct2/show/NCT04952727.

[187] Ghasemiyeh P Mohammadi-Samani S Firouzabadi N Dehshahri A Vazin A. A Focused Review on Technologies, Mechanisms, Safety, and Efficacy of Available COVID-19 Vaccines. Int Immunopharmacol (2021) 100:108162. doi: 10.1016/j.intimp.2021.108162 34562844 PMC8445802

[188] A Study of Ad26.COV2.S for the Prevention of SARS-CoV-2-Mediated COVID-19 in Adult Participants (ENSEMBLE). NIH (2020). Available at: https://clinicaltrials.gov/ct2/show/NCT04505722.

[189] A Study of Ad26.COV2.S for the Prevention of SARS-CoV-2-Mediated COVID-19 in Adults (ENSEMBLE 2). NIH (2020). Available at: https://clinicaltrials.gov/ct2/show/NCT04614948.

[190] Sisonke (Together): OPEN LABEL TRIAL COVID-19 (Sisonke). NIH (2021). Available at: https://clinicaltrials.gov/ct2/show/NCT04838795.

[191] Allergy and COVID-19 Vaccines (COVALL). NIH (2021). Available at: https://clinicaltrials.gov/ct2/show/NCT05028257.

[192] RECOVAC Booster Vaccination Study. NIH (2021). Available at: https://clinicaltrials.gov/ct2/show/NCT05030974.

[193] Corchado-Garcia J Puyraimond-Zemmour D Hughes T Cristea-Platon T Lenehan P Pawlowski C. Real-World Effectiveness of Ad26.COV2.S Adenoviral Vector Vaccine for COVID-19. medRxiv (2021) 2021:04.27.21256193. doi: 10.1101/2021.04.27.21256193 PMC8564583 34726743

[194] Logunov DY Dolzhikova IV Shcheblyakov DV Tukhvatulin AI Zubkova OV Dzharullaeva AS. Safety and Efficacy of an Rad26 and Rad5 Vector-Based Heterologous Prime-Boost COVID-19 Vaccine: An Interim Analysis of a Randomised Controlled Phase 3 Trial in Russia. Lancet (2021) 397:671–81. doi: 10.1016/S0140-6736(21)00234-8 PMC7852454 33545094

[195] Tsimafeyeu I Volkova M Alekseeva G Berkut M Nosov A Myslevtsev I. Safety and Preliminary Efficacy of the Gam-COVID-Vac Vaccine and Outcomes of SARS-CoV-2 Infection in Russian Patients With Genitourinary Malignancies. J Hematol Oncol (2021) 14:192. doi: 10.1186/s13045-021-01205-z 34774086 PMC8590125

[196] Montalti M Solda G Di Valerio Z Salussolia A Lenzi J Forcellini M. ROCCA Observational Study: Early Results on Safety of Sputnik V Vaccine (Gam-COVID-Vac) in the Republic of San Marino Using Active Surveillance. EClinicalMedicine (2021) 38:101027. doi: 10.1016/j.eclinm.2021.101027 34505029 PMC8413252

[197] Gonzalez S Olszevicki S Salazar M Calabria A Regairaz L Marin L. Effectiveness of the First Component of Gam-COVID-Vac (Sputnik V) on Reduction of SARS-CoV-2 Confirmed Infections, Hospitalisations and Mortality in Patients Aged 60-79: A Retrospective Cohort Study in Argentina. EClinicalMedicine (2021) 40:101126. doi: 10.1016/j.eclinm.2021.101126 34541480 PMC8435263

[198] Clinical Trial of Efficacy, Safety, and Immunogenicity of Gam-COVID-Vac Vaccine Against COVID-19 (RESIST). NIH (2021). Available at: https://clinicaltrials.gov/ct2/show/NCT04530396.

[199] Clinical Trial of Efficacy, Safety, and Immunogenicity of Gam-COVID-Vac Vaccine Against COVID-19 in Belarus. NIH (2021). Available at: https://clinicaltrials.gov/ct2/show/NCT04564716.

[200] Clinical Trial of the Immunogenicity, Safety, and Efficacy of the Gam-COVID-Vac Vaccine Against COVID-19 in Venezuela (VENEZUELA). NIH (2021). Available at: https://clinicaltrials.gov/ct2/show/NCT04642339.

[201] A Phase III Clinical Trial of the Immunogenicity and Safety of the Gam-COVID-Vac Vaccine Against COVID-19 in the UAE (SPUTNIK-UAE). NIH (2021). Available at: https://clinicaltrials.gov/ct2/show/NCT04656613.

[202] Tukhvatulin AI Dolzhikova IV Shcheblyakov DV Zubkova OV Dzharullaeva AS Kovyrshina AV. An Open, non-Randomised, Phase 1/2 Trial on the Safety, Tolerability, and Immunogenicity of Single-Dose Vaccine "Sputnik Light" for Prevention of Coronavirus Infection in Healthy Adults. Lancet Regional Health Europe (2021) 11:100241. doi: 10.1016/j.lanepe.2021.100241 PMC8562788 34746910

[203] Study to Evaluate Efficacy, Immunogenicity and Safety of the Sputnik-Light (SPUTNIK-LIGHT). NIH (2021). Available at: https://clinicaltrials.gov/ct2/show/NCT04741061.

[204] A Phase III, Randomized, Double Blind, Placebo-Controlled International Multisite Clinical Trial in Parallel Assignment to Evaluate Efficacy, Immunogenicity and Safety of the Sputnik Light Vector Vaccine in Adults in the SARS-CoV-2 Infection Prophylactic Treatment. COVID-19. Pan Afr Clin Trials Registry (2021).

[205] Mallapaty S. WHO Approval of Chinese CoronaVac COVID Vaccine Will be Crucial to Curbing Pandemic. Nature (2021) 594:161–2. doi: 10.1038/d41586-021-01497-8 34089030

[206] Mallapaty S. China’s COVID Vaccines are Going Global-But Questions Remain. Nature (2021) 593:178–9. doi: 10.1038/d41586-021-01146-0 33948031

[207] Vacharathit V Aiewsakun P Manopwisedjaroen S Srisaowakarn C Laopanupong T Ludowyke N. CoronaVac Induces Lower Neutralising Activity Against Variants of Concern Than Natural Infection. Lancet Infect Dis (2021) 21:1352–4. doi: 10.1016/S1473-3099(21)00568-5 PMC8389976 34454652

[208] Royal A Ahmad S Qureshi A Chaudhary V Jindal T Kumar V. An Altmetric Analysis of Online News on India’s First Indigenous COVID-19 Vaccine. J Educ Health Promotion (2021) 10:348. doi: 10.4103/jehp.jehp_1603_20 PMC8552286 34761034

[209] How India is Responding to COVID-19: Quarantine, Travel Limits and Tests (2020). Available at: https://www.weforum.org/agenda/2020/03/quarantine-india-covid-19-coronavirus/

[210] Raina SK Kumar R. "Covishield and Covaxin" - India’s Contribution to Global COVID-19 Pandemic. J Family Med Primary Care (2021) 10:2433–5. doi: 10.4103/jfmpc.jfmpc_174_21 PMC8415646 34568116

[211] Kumar NP Banurekha VV Gerish kumar CP Nancy A Padmapriyadarsini C Mary AS. Prime-Boost Vaccination With Covaxin/BBV152 Induces Heightened Systemic Cytokine and Chemokine Responses. Front Immunol (2021) 12:752397. doi: 10.3389/fimmu.2021.752397 34721425 PMC8554328

[212] WHO Newsroom. The Different Types of COVID-19 Vaccines (2021). Available at: https://www.who.int/news-room/feature-stories/detail/the-race-for-a-covid-19-vaccine-explained.

[213] Cunningham AL Garcon N Leo O Friedland LR Strugnell R Laupeze B. Vaccine Development: From Concept to Early Clinical Testing. Vaccine (2016) 34:6655–64. doi: 10.1016/j.vaccine.2016.10.016 27769596

[214] Arnon R Ben-Yedidia T. Old and New Vaccine Approaches. Int Immunopharmacol (2003) 3:1195–204. doi: 10.1016/S1567-5769(03)00016-X 12860175

[215] Holm MR Poland GA. Critical Aspects of Packaging, Storage, Preparation, and Administration of mRNA and Adenovirus-Vectored COVID-19 Vaccines for Optimal Efficacy. Vaccine (2021) 39:457–9. doi: 10.1016/j.vaccine.2020.12.017 PMC7723768 33339671

[216] Vogel AB Lambert L Kinnear E Busse D Erbar S Reuter KC. Self-Amplifying RNA Vaccines Give Equivalent Protection Against Influenza to mRNA Vaccines But at Much Lower Doses. Mol Ther (2018) 26:446–55. doi: 10.1016/j.ymthe.2017.11.017 PMC5835025 29275847

[217] Pardi N Hogan MJ Porter FW Weissman D. mRNA Vaccines - a New Era in Vaccinology. Nat Rev Drug Discov (2018) 17:261–79. doi: 10.1038/nrd.2017.243 PMC5906799 29326426

[218] Brito LA Kommareddy S Maione D Uematsu Y Giovani C Berlanda Scorza F. Self-Amplifying mRNA Vaccines. Adv Genet (2015) 89:179–233. doi: 10.1016/bs.adgen.2014.10.005 25620012

[219] Rauch S Jasny E Schmidt KE Petsch B. New Vaccine Technologies to Combat Outbreak Situations. Front Immunol (2018) 9:1963. doi: 10.3389/fimmu.2018.01963 30283434 PMC6156540

[220] Saxena S Sonwane AA Dahiya SS Patel CL Saini M Rai A. Induction of Immune Responses and Protection in Mice Against Rabies Using a Self-Replicating RNA Vaccine Encoding Rabies Virus Glycoprotein. Vet Microbiol (2009) 136:36–44. doi: 10.1016/j.vetmic.2008.10.030 19081687

[221] Zhang C Maruggi G Shan H Li J. Advances in mRNA Vaccines for Infectious Diseases. Front Immunol (2019) 10:594. doi: 10.3389/fimmu.2019.00594 30972078 PMC6446947

[222] Fomsgaard A Liu MA. The Key Role of Nucleic Acid Vaccines for One Health. Viruses (2021) 13. doi: 10.3390/v13020258 PMC7916035 33567520

[223] Yi C Yi Y Li J. mRNA Vaccines: Possible Tools to Combat SARS-CoV-2. Virol Sin (2020) 35:259–62. doi: 10.1007/s12250-020-00243-0 PMC7286219 32524253

[224] Hall VG Ferreira VH Ku T Ierullo M Majchrzak-Kita B Chaparro C. Randomized Trial of a Third Dose of mRNA-1273 Vaccine in Transplant Recipients. New Engl J Med (2021) 385:1244–6. doi: 10.1056/NEJMc2111462 PMC8385563 34379917

[225] Sahin U Muik A Derhovanessian E Vogler I Kranz LM Vormehr M. Publisher Correction: COVID-19 Vaccine BNT162b1 Elicits Human Antibody and TH1 T Cell Responses. Nature (2021) 590:E17. doi: 10.1038/s41586-020-03102-w 33469214

[226] Dagan N Barda N Kepten E Miron O Perchik S Katz MA. BNT162b2 mRNA Covid-19 Vaccine in a Nationwide Mass Vaccination Setting. New Engl J Med (2021) 384:1412–23. doi: 10.1056/NEJMoa2101765 PMC7944975 33626250

[227] Rauch S Roth N Schwendt K Fotin-Mleczek M Mueller SO Petsch B. mRNA Based SARS-CoV-2 Vaccine Candidate CVnCoV Induces High Levels of Virus Neutralizing Antibodies and Mediates Protection in Rodents. BioRxiv Preprint Server Biol (2021) 6:57. doi: 10.1038/s41541-021-00311-w PMC8052455 33863911

[228] Roth N Schön J Hoffmann D Thran M Thess A Mueller SO. CV2CoV, an Enhanced mRNA-Based SARS-CoV-2 Vaccine Candidate, Supports Higher Protein Expression and Improved Immunogenicity in Rats. BioRxiv Preprint Server Biol (2021) 2021:05.13.443734. doi: 10.1101/2021.05.13.443734

[229] Second-Generation mRNA-Based COVID-19 Vaccine Candidate, CV2CoV, Shows Improved Immune Response and Protective Effect in Preclinical Study. Pharmiweb (2021). Available at: https://www.pharmiweb.com/press-release/2021-08-17/second-generation-mrna-based-covid-19-vaccine-candidate-cv2cov-shows-improved-immune-response-and-protective-effect-in-preclinical-study

[230] Kremsner PG Guerrero RAA Arana-Arri E Martinez GJA Bonten M Chandler R. Efficacy and Safety of the CVnCoV SARS-CoV-2 mRNA Vaccine Candidate: Results From Herald, a Phase 2b/3, Randomised, Observer-Blinded, Placebo-Controlled Clinical Trial in Ten Countries in Europe and Latin America(2021). Available at: https://ssrn.com/abstract=3911826.

[231] Schlake T Thess A Fotin-Mleczek M Kallen K-J. Developing mRNA-Vaccine Technologies. RNA Biol (2012) 9:1319–30. doi: 10.4161/rna.22269 PMC3597572 23064118

[232] Morais P Adachi H Yu Y-T. The Critical Contribution of Pseudouridine to mRNA COVID-19 Vaccines. Front Cell Dev Biol (2021) 9:789427–. doi: 10.3389/fcell.2021.789427 PMC8600071 34805188

[233] Jeeva S Kim K-H Shin CH Wang B-Z Kang S-M. An Update on mRNA-Based Viral Vaccines. Vaccines (2021) 9:965. doi: 10.3390/vaccines9090965 34579202 PMC8473183

[234] Verbeke R Lentacker I De Smedt SC Dewitte H. The Dawn of mRNA Vaccines: The COVID-19 Case. J Controlled Release (2021) 333:511–20. doi: 10.1016/j.jconrel.2021.03.043 PMC8008785 33798667

[235] Aldosari BN Alfagih IM Almurshedi AS. Lipid Nanoparticles as Delivery Systems for RNA-Based Vaccines. Pharmaceutics (2021) 13:206. doi: 10.3390/pharmaceutics13020206 33540942 PMC7913163

[236] Wadhwa A Aljabbari A Lokras A Foged C Thakur A. Opportunities and Challenges in the Delivery of mRNA-Based Vaccines. Pharmaceutics (2020) 12. doi: 10.3390/pharmaceutics12020102 PMC7076378 32013049

[237] Tsilingiris D Vallianou NG Karampela I Liu J Dalamaga M. Potential Implications of Lipid Nanoparticles in the Pathogenesis of Myocarditis Associated With the Use of mRNA Vaccines Against SARS-CoV-2. Metabol Open (2022) 13:100159–. doi: 10.1016/j.metop.2021.100159 PMC8677426 34938983

[238] Plotkin SA. Vaccines: Past, Present and Future. Nat Med (2005) 11:S5–11. doi: 10.1038/nm1209 15812490 PMC7095920

[239] Naz SS Aslam A Malik T. An Overview of Immune Evasion Strategies of DNA and RNA Viruses. Infect Disord Drug Targets (2021) 21:e300821192322. doi: 10.2174/1871526521666210317161329 33739247

[240] Heppell J Davis HL. Application of DNA Vaccine Technology to Aquaculture. Adv Drug Deliv Rev (2000) 43:29–43. doi: 10.1016/S0169-409X(00)00075-2 10967219

[241] Rappuoli R Mandl CW Black S De Gregorio E. Vaccines for the Twenty-First Century Society. Nat Rev Immunol (2011) 11:865–72. doi: 10.1038/nri3085 PMC7098427 22051890

[242] Yang ZY Kong WP Huang Y Roberts A Murphy BR Subbarao K. A DNA Vaccine Induces SARS Coronavirus Neutralization and Protective Immunity in Mice. Nature (2004) 428:561–4. doi: 10.1038/nature02463 PMC7095382 15024391

[243] Lee P Kim CU Seo SH Kim DJ. Current Status of COVID-19 Vaccine Development: Focusing on Antigen Design and Clinical Trials on Later Stages. Immune Netw (2021) 21:e4. doi: 10.4110/in.2021.21.e4 33728097 PMC7937514

[244] Lopez-Sagaseta J Malito E Rappuoli R Bottomley MJ. Self-Assembling Protein Nanoparticles in the Design of Vaccines. Comput Struct Biotechnol J (2016) 14:58–68. doi: 10.1016/j.csbj.2015.11.001 26862374 PMC4706605

[245] Speiser DE Bachmann MF. COVID-19: Mechanisms of Vaccination and Immunity. Vaccines (2020) 8. doi: 10.3390/vaccines8030404 PMC7564472 32707833

[246] Jones KL Drane D Gowans EJ. Long-Term Storage of DNA-Free RNA for Use in Vaccine Studies. BioTechniques (2007) 43:675–81. doi: 10.2144/000112593 PMC4526277 18072597

[247] Shih HI Wu CJ Tu YF Chi CY. Fighting COVID-19: A Quick Review of Diagnoses, Therapies, and Vaccines. Biomed J (2020) 43:341–54. doi: 10.1016/j.bj.2020.05.021 PMC7260535 32532623

[248] Smith TRF Patel A Ramos S Elwood D Zhu X Yan J. Immunogenicity of a DNA Vaccine Candidate for COVID-19. Nat Commun (2020) 11:2601. doi: 10.1038/s41467-020-16505-0 32433465 PMC7239918

[249] Dey A Chozhavel Rajanathan TM Chandra H Pericherla HPR Kumar S Choonia HS. Immunogenic Potential of DNA Vaccine Candidate, ZyCoV-D Against SARS-CoV-2 in Animal Models. Vaccine (2021) 39:4108–16. doi: 10.1016/j.vaccine.2021.05.098 PMC8166516 34120764

[250] Cao Q Wu S Xiao C Chen S Chi X Cui X. Integrated Single-Cell Analysis Revealed Immune Dynamics During Ad5-Ncov Immunization. Cell Discov (2021) 7:64. doi: 10.1038/s41421-021-00300-2 34373443 PMC8352953

[251] Hernandez-Bello J Morales-Nunez JJ Machado-Sulbaran AC Diaz-Perez SA Torres-Hernandez PC Balcazar-Felix P. Neutralizing Antibodies Against SARS-CoV-2, Anti-Ad5 Antibodies, and Reactogenicity in Response to Ad5-Ncov (CanSino Biologics) Vaccine in Individuals With and Without Prior SARS-CoV-2. Vaccines (2021) 9. doi: 10.3390/vaccines9091047 PMC8472849 34579284

[252] Zhu FC Guan XH Li YH Huang JY Jiang T Hou LH. Immunogenicity and Safety of a Recombinant Adenovirus Type-5-Vectored COVID-19 Vaccine in Healthy Adults Aged 18 Years or Older: A Randomised, Double-Blind, Placebo-Controlled, Phase 2 Trial. Lancet (2020) 396:479–88. doi: 10.1016/S0140-6736(20)31605-6 PMC7836858 32702299

[253] Aguas R Bharath A White LJ Gao B Pollard AJ Voysey M. Potential Global Impacts of Alternative Dosing Regimen and Rollout Options for the ChAdOx1 Ncov-19 Vaccine. Nat Commun (2021) 12:6370. doi: 10.1038/s41467-021-26449-8 34737262 PMC8569205

[254] Folegatti PM Ewer KJ Aley PK Angus B Becker S Belij-Rammerstorfer S. Safety and Immunogenicity of the ChAdOx1 Ncov-19 Vaccine Against SARS-CoV-2: A Preliminary Report of a Phase 1/2, Single-Blind, Randomised Controlled Trial. Lancet (2020) 396:467–78. doi: 10.1016/S0140-6736(20)31604-4 PMC7445431 32702298

[255] van Doremalen N Lambe T Spencer A Belij-Rammerstorfer S Purushotham JN Port JR. ChAdOx1 Ncov-19 Vaccine Prevents SARS-CoV-2 Pneumonia in Rhesus Macaques. Nature (2020) 586:578–82. doi: 10.1038/s41586-020-2608-y PMC8436420 32731258

[256] Ewer KJ Barrett JR Belij-Rammerstorfer S Sharpe H Makinson R Morter R. T Cell and Antibody Responses Induced by a Single Dose of ChAdOx1 Ncov-19 (AZD1222) Vaccine in a Phase 1/2 Clinical Trial. Nat Med (2021) 27:270–8. doi: 10.1038/s41591-020-01194-5 33335323

[257] Bos R Rutten L van der Lubbe JEM Bakkers MJG Hardenberg G Wegmann F. Ad26 Vector-Based COVID-19 Vaccine Encoding a Prefusion-Stabilized SARS-CoV-2 Spike Immunogen Induces Potent Humoral and Cellular Immune Responses. NPJ Vaccines (2020) 5:91. doi: 10.1038/s41541-020-00243-x 33083026 PMC7522255

[258] Sablerolles RSG Rietdijk WJR Goorhuis A Postma DF Visser LG Geers D. Immunogenicity and Reactogenicity of Booster Vaccinations After Ad26.COV2.S Priming. medRxiv (2021) 386:951–963. doi: 10.1056/NEJMoa2116747 PMC8796791 35045226

[259] Solforosi L Kuipers H Jongeneelen M Rosendahl Huber SK van der Lubbe JEM Dekking L. Immunogenicity and Efficacy of One and Two Doses of Ad26.COV2.S COVID Vaccine in Adult and Aged NHP. J Exp Med (2021) 218. 10.1084/jem.20202756 PMC8085771 33909009

[260] Anywaine Z Whitworth H Kaleebu P Praygod G Shukarev G Manno D. Safety and Immunogenicity of a 2-Dose Heterologous Vaccination Regimen With Ad26.ZEBOV and MVA-BN-Filo Ebola Vaccines: 12-Month Data From a Phase 1 Randomized Clinical Trial in Uganda and Tanzania. J Infect Dis (2019) 220:46–56. doi: 10.1093/infdis/jiz070 30796818 PMC6548900

[261] Williams K Bastian AR Feldman RA Omoruyi E de Paepe E Hendriks J. Phase 1 Safety and Immunogenicity Study of a Respiratory Syncytial Virus Vaccine With an Adenovirus 26 Vector Encoding Prefusion F (Ad26.RSV.preF) in Adults Aged >/=60 Years. J Infect Dis (2020) 222:979–88. doi: 10.1093/infdis/jiaa193 32320465

[262] Nogrady B. Mounting Evidence Suggests Sputnik COVID Vaccine is Safe and Effective. Nature (2021) 595:339–40. doi: 10.1038/d41586-021-01813-2 34230663

[263] Jones I Roy P. Sputnik V COVID-19 Vaccine Candidate Appears Safe and Effective. Lancet (2021) 397:642–3. doi: 10.1016/S0140-6736(21)00191-4 PMC7906719 33545098

[264] Naygovzina NB Khabriev RU Krasheninnikov AE Matveev AV. [The Organizational Aspects of Security Support of Participants of Clinical Testing of Vaccine "Gam-COVID-Vac’]. Problemy Sotsial’noi Gigieny Zdravookhraneniia i Istorii Meditsiny (2021) 11:587615. doi: 10.3389/fimmu.2020.587615 33591649

